# Uncovering Social States in Healthy and Clinical Populations Using Digital Phenotyping and Hidden Markov Models: Observational Study

**DOI:** 10.2196/64007

**Published:** 2025-04-28

**Authors:** Imogen E Leaning, Andrea Costanzo, Raj Jagesar, Lianne M Reus, Pieter Jelle Visser, Martien J H Kas, Christian F Beckmann, Henricus G Ruhé, Andre F Marquand

**Affiliations:** 1 Donders Institute for Brain, Cognition and Behaviour Radboud University Nijmegen Nijmegen The Netherlands; 2 Department for Medical Neuroscience Radboud University Medical Center Nijmegen Nijmegen The Netherlands; 3 Groningen Institute for Evolutionary Life Sciences University of Groningen Groningen The Netherlands; 4 Department of Neurology, Alzheimer Center Amsterdam Neuroscience Amsterdam UMC Amsterdam The Netherlands; 5 Amsterdam Neuroscience, Neurodegeneration Amsterdam UMC Amsterdam The Netherlands; 6 Center for Neurobehavioral Genetics, Semel Institute for Neuroscience and Human Behavior David Geffen School of Medicine University of California Los Angeles, CA United States; 7 Department of Psychiatry & Neuropsychology School for Mental Health and Neuroscience Maastricht University Maastricht The Netherlands; 8 Department of Neurobiology, Care Sciences and Society Division of Neurogeriatrics Karolinska Institutet Stockholm Sweden; 9 Department of Psychiatry Radboud University Medical Center Nijmegen Nijmegen The Netherlands; 10 Department of Neuroimaging Institute of Psychiatry, Psychology and Neuroscience King’s College London London United Kingdom

**Keywords:** passive monitoring, mobile health, mHealth, smartphone, mobile phone, digital phenotyping, hidden Markov model, social behavior, Alzheimer disease, cognitive impairment, schizophrenia

## Abstract

**Background:**

Brain-related disorders are characterized by observable behavioral symptoms, for example, social withdrawal. Smartphones can passively collect behavioral data reflecting digital activities such as communication app usage and calls. These data are collected objectively in real time, avoiding recall bias, and may, therefore, be a useful tool for measuring behaviors related to social functioning. Despite promising clinical utility, analyzing smartphone data is challenging as datasets often include a range of temporal features prone to missingness.

**Objective:**

Hidden Markov models (HMMs) provide interpretable, lower-dimensional temporal representations of data, allowing for missingness. This study aimed to investigate the HMM as a method for modeling smartphone time series data.

**Methods:**

We applied an HMM to an aggregate dataset of smartphone measures designed to assess phone-related social functioning in healthy controls (HCs) and participants with schizophrenia, Alzheimer disease (AD), and memory complaints. We trained the HMM on a subset of HCs (91/348, 26.1%) and selected a model with socially *active* and *inactive* states. Then, we generated hidden state sequences per participant and calculated their “total dwell time,” that is, the percentage of time spent in the socially active state. Linear regression models were used to compare the total dwell time to social and clinical measures in a subset of participants with available measures, and logistic regression was used to compare total dwell times between diagnostic groups and HCs. We primarily reported results from a 2-state HMM but also verified results in HMMs with more hidden states and trained on the whole participant dataset.

**Results:**

We identified lower total dwell times in participants with AD (26/257, 10.1%) versus withheld HCs (156/257, 60.7%; odds ratio 0.95, 95% CI 0.92-0.97; false discovery rate [FDR]–corrected *P*<.001), as well as in participants with memory complaints (57/257, 22.2%; odds ratio 0.97, 95% CI 0.96-0.99; FDR-corrected *P*=.004). The result in the AD group was very robust across HMM variations, whereas the result in the memory complaints group was less robust. We also observed an interaction between the AD group and total dwell time when predicting social functioning (FDR-corrected *P*=.02). No significant relationships regarding total dwell time were identified for participants with schizophrenia (18/257, 7%; *P*>.99).

**Conclusions:**

We found the HMM to be a practical, interpretable method for digital phenotyping analysis, providing an objective phenotype that is a possible indicator of social functioning.

## Introduction

### Background

Many psychiatric and neurological diseases exhibit observable behaviors that indicate the underlying condition. For example, social functioning is negatively impacted in a broad range of conditions, including schizophrenia, major depressive disorder, anxiety disorders, and Alzheimer disease (AD) [1-3], often cumulating in social withdrawal. Social withdrawal, indicated by reduced social interaction [1], can be observed as people engage less with those around them. However, successfully measuring behavioral components such as social withdrawal is challenging, as reports of behavior are subjective and susceptible to recall bias, with questionnaires often being burdensome to complete. Therefore, there is a need to develop practical, objective tools to monitor these symptoms, for example, to predict or measure clinically relevant changes.

The field of digital phenotyping is developing to meet such a need. Digital phenotyping involves the development of behavioral or physiological markers calculated from digital measures. “Digital phenotype” is a broad term referring to a quantified digital behavior (such as the use of smartphone apps) or behavior measured using a digital signal (such as movement measured using GPS). These measures avoid issues of recall bias as they are objective and can be acquired in real time as participants go about their day, meaning they have high ecological validity. A popular tool to collect digital phenotyping data is the smartphone. Given how commonplace smartphones are in society, they are a convenient data collection tool as they do not require participants to change their behavior or routines. A monitoring app, for example, “Behapp” [4], “Mood mirror” [5], or “RADAR-base pRMT” [6], can be installed on the participants’ smartphones and run passively in the background to collect data without user intervention.

Modern smartphones have many sensors and functionalities, including various apps, calling capabilities, Wi-Fi, GPS, accelerometer, and Bluetooth, which can be leveraged to model different aspects of behavior, such as social contacts, movement patterns, and app usage [7,8]. Many of these data streams are direct measures of digital behaviors that can be used as proxy measures of social behavior; for example, the use of communication apps could indicate how connected someone is with their contacts. While using these measures requires inferences to be made about behavior, their objective nature and the range in available measures means they are a promising tool for modeling social behavior.

Moreover, there are many ways in which these data can be processed. For example, duration, rhythm, or statistical measures can be calculated (such as daily durations of a behavior, circadian rhythm, or mean and SD of a behavior across time), or the occurrences of the behavior can be counted [9]. This often leads to datasets with many features reflecting various smartphone-measured behaviors. A major problem affecting digital phenotyping is that data collection platforms are often prone to missing data due to the difficulties of real-world longitudinal data collection, leading to missing values across all or a subset of these features [9].

The issues and complexities observed in digital phenotyping research give rise to multiple analytic challenges. Processing the collected feature sets, often representing a wide range of seemingly distinct observed behaviors with potentially similar underlying causes, requires many model decisions. Therefore, appropriate methods are needed to analyze this multifaceted data containing missing values to produce meaningful, lower-dimensional data representations. These representations may be more usable and informative about the underlying behavioral states of participants than the individual features. Models should also aim to be interpretable not only by researchers but also by clinicians and patients to facilitate their use in clinical practice. A further property that would enable their use in this context is that they can preserve the time domain, as one of the goals of smartphone digital phenotyping is to be able to make useful clinical predictions that can enable early intervention. Many digital phenotyping studies have focused on time-averaged features and analyses, and a shift toward more direct investigations of temporal dynamics is expected to improve clinical utility [9].

In addition, given the range of symptoms experienced by people with various neuropsychiatric disorders, it may be useful to define a reference distribution that could represent a “standard operating range” for a given population or participant, where deviations from this range can then be conceptualized as signaling transitions into different behavioral modes of functioning, as is done in normative modeling [10,11] or anomaly detection applications [12,13]. This reference distribution could be, for example, data from healthy controls (HCs) or from periods when individuals are not experiencing a relapse of their disorder. This approach may also help to leverage more easily collectable periods of data, as it can be challenging to capture periods containing relapses or the symptom severity range that is of interest, leading to smaller volumes of data for these periods.

Currently, digital phenotyping studies use a broad range of modeling approaches, for example, investigating associations between neuropsychiatric symptoms and summary measures (eg, total number of places visited and mean duration of communication app usage) [14]; clustering of digital phenotypes to investigate transdiagnostic symptom classification [15]; linear mixed effects models accounting for repeated measures of time-averaged features [16-19]; multivariate anomaly detection to identify relapse in schizophrenia [20]; and joinpoint regression to identify changes in the trajectory of digital phenotypes (eg, step count) [21].

### This Study

In this study, we propose the use of a hidden Markov model (HMM) [22] as a method to model digital phenotyping time series data. This model provides several appealing features, namely, HMMs (1) can meaningfully combine different behavioral features, (2) reflect changes in behavior over time, (3) provide readily interpretable summary statistics, and (4) naturally accommodate missingness. HMMs provide interpretable, lower-dimensional representations of the data using latent (ie, hidden) states, where the observed time series channels are represented as a sequence of these hidden states. Each hidden state has associated “emission probabilities” indicating the probability that a set of observed behaviors occurs when the sequence is in the said hidden state, allowing for informative behavioral states to be derived by representing >1 feature per state. Changes in behavior through time are modeled via transitions between these hidden states. Importantly for digital phenotyping, HMMs contain intrinsic mechanisms for handling missing data. HMMs have been used in many applications for modeling behavior, for example, to model drinking patterns in people with an alcohol use disorder [23], cocaine dependence [24], sleep patterns represented in neuroimaging data [25], mobility data [26,27], weekly psychotic depressive symptom profiles [28], weekly depressive symptom profiles [29], and actigraphy and survey data reflecting behavior and affect in college students [30].

While our approach is widely applicable to digital phenotyping time series, in this work, we demonstrate its application to data collected using the Behapp monitoring app [31], which collects passive data related to app usage, calls, GPS, Wi-Fi, and overall phone usage, reflecting the periods the phone was unlocked. We applied an HMM to a combined dataset of phone usage and communication-related features from participants in the “Psychiatric Ratings using Intermediate Stratified Markers” (PRISM) [32] and Hersenonderzoek [14] studies, demonstrating how an HMM can successfully represent digital phenotyping time series. The model was initially trained on a set of HCs with low missingness to provide a high-quality dataset for training, which was treated as a “reference category.” The trained model was then applied to HCs with higher missingness, participants with AD and schizophrenia, and healthy participants with memory complaints (subjective cognitive complaints [SCC]) to investigate the applicability of such a model to clinical groups and participants with lower data availability. Hidden state sequences were generated for these participants, and we then calculated a digital phenotype derived from the HMM for each participant, namely the “total dwell time.” Rather than being a directly observed digital phenotype (such as the percentage of time spent using communication apps), the total dwell time provides the percentage of time the participant spent in a hidden behavioral state derived from the observed digital measures. This digital phenotype was then linked to clinical measures, including diagnostic group and social functioning, demonstrating the clinical value of this approach.

## Methods

### Participants

#### Overview

This analysis used data from participants from the PRISM and Hersenonderzoek studies. We chose to combine these datasets in our analysis due to the overlap in populations, as both studies included participants with AD and, consequently, similarly age-matched HCs, meaning we could have an increased sample size for the AD and HC groups.

#### PRISM Study

The PRISM study aimed to investigate social withdrawal in 2 brain disorders, schizophrenia and probable AD [32,33]. Participants with AD, participants with schizophrenia, and age- and sex-matched HCs were recruited across centers in Spain (Hospital General Universitario Gregorio Marañón and Hospital Universitario de La Princesa, Madrid) and the Netherlands (University Medical Center Utrecht, Leiden University Medical Center, and Amsterdam University Medical Center [location Vrije Universiteit Medical Center]).

Participants with schizophrenia were required to be within the age range of 18 to 45 years (inclusive) and to have a *Diagnostic and Statistical Manual of Mental Disorders-IV* diagnosis of schizophrenia confirmed by the Mini-International Neuropsychiatric Interview. Participants were required to have experienced at least 1 psychotic episode, to have had a maximum disease duration of 10 years since diagnosis, and for any antipsychotic medication dosage to have been stable for a minimum of 8 weeks. As PRISM aimed to investigate social withdrawal linked with negative symptoms (and not because of other sources such as psychosis), participants with schizophrenia were excluded if they rated highly for positive symptoms (≥22 on the positive symptom factor of the 7-item Positive and Negative Syndrome Scale [PANSS]) [34]. A positive symptom indicates an additional experience an individual is having, such as a hallucination or delusion, as opposed to a negative symptom, which indicates a deficit in an already existing function, such as a deficit in concentration. While schizophrenia is commonly associated with positive symptoms, negative symptoms also form a large component of the disorder. Participants with AD were required to be within the age range of 50 to 80 years, to meet the classification of “probable AD” based on the National Institute on Aging and the Alzheimer’s Association criteria, and to have a Mini-Mental State Examination (MMSE) [35] score of 20 to 26. For both participants with schizophrenia and AD, it was required that participants were not socially withdrawn due to other reasons, such as their external circumstances or a comorbid medical disorder or disability. These factors were evaluated during the intake interview.

HCs were recruited in the age ranges of 18 to 45 years and 50 to 80 years and were required to have an approximately average MMSE score according to their age and years of education. Participants were excluded if they met the criteria for an Axis-I psychiatric disorder (assessed by the Mini-International Neuropsychiatric Interview) or a neurological disease associated with cognitive impairment. The PRISM study overview by Bilderbeck et al [32] provides further details of inclusion and exclusion criteria for all participant groups.

In addition to Behapp data collection, measures of clinical and social functioning were acquired. The self-report Social Functioning Scale (SFS) [36] and the De Jong Gierveld Loneliness and Affiliation Scale [37] were administered to all participants, the MMSE was administered to HCs and participants with AD, and the PANSS was administered to participants with schizophrenia.

#### Hersenonderzoek Study

Participants with probable AD, SCC, and age-matched HCs were recruited across the Netherlands by the Dutch Brain Research Registry [38], providing demographics and health-related information on the web via the Hersenonderzoek platform [14]. Participants indicated the presence of probable AD. To classify participants as those with SCC or HCs, participants indicated that they had an absence of neurological or psychiatric diseases, either with or without memory complaints, respectively. The minimum age for inclusion was 45 years.

### Ethical Considerations

PRISM was approved by the Ethical Review Board University Medical Centre of Utrecht (17-021/D) for the participating research centers in the Netherlands and by the Comité Ético de Investigación Clínica Hospital General Universitario Gregorio Marañón (59359) for the participating research centers in Spain. PRISM participants were deemed by the researcher and caregivers to be sufficiently competent to participate in the study. Approval for Hersenonderzoek was provided by the Ethical Review Board VU University Medical Centre (2017.254). All PRISM and Hersenonderzoek participants provided informed consent before participation commenced. In the PRISM study, participants received both travel expenses and compensation for their time. For the Hersenonderzoek study, it was possible to receive travel expenses. In both studies, participants’ data were deidentified. Participants could request the deletion of their collected data from the database at any time, in line with the General Data Protection Regulation.

### Behapp Acquisition

The smartphone app, “Behapp” [31], was installed on participants’ smartphones. Behapp passively collected smartphone usage data for 42 days without storing any content of messages and calls, in compliance with the European Privacy Regulation [39]. The classification of each app used by participants was gathered from the Google Play Store, so that apps could be grouped by type, including social media and communication apps. During the time of data collection (PRISM: August 2017 to May 2019 and Hersenonderzoek: March 2018 to January 2020), Behapp was only available on Android smartphones; therefore, PRISM participants who did not have their own Android smartphone were supplied with one for the duration of study participation. However, this was not done for Hersenonderzoek participants in accordance with the study design, and only 2 PRISM participants used a study-provided phone. For each activity (eg, use of an app), the respective start and end timestamps were stored.

### Preprocessing

#### Smartphone Channels

Phone usage was split into 5 categories, referred to as “channels”: social media app usage, communication app usage, incoming calls, outgoing calls, and overall phone usage. GPS channels were also available. Since many of these measures were sparsely sampled, each channel was aggregated into hourly bins, and the percentage of each hour for which each activity was carried out was calculated. For example, a participant may spend 100% of an hour using their phone, 50% on social media, 40% on communication apps, 0% making or receiving calls, and 10% using another functionality, such as Google Maps. Even with the temporal resampling, many of these phenotypes have highly zero-inflated distributions (Figure S1 in Multimedia Appendix 1), which can be difficult to handle natively. Therefore, for each hourly time point, these percentages were grouped into discrete bins instead of continuous percentages such as binary bins reflecting either no or some activity carried out in the hour (0% activity or >0% activity). We chose this low threshold to define activity, as many of the activities we investigated may still be meaningful despite their short duration, for example, the time it took to send a message. We conducted a sensitivity analysis to understand the impact that this activity threshold had and found that a threshold requiring an activity to be carried out for at least 5% of an hour provided comparable results to the HMMs presented here; however, this was no longer the case for a 10% threshold. With this threshold, very few hours were classified as containing activity (Figure S1 in Multimedia Appendix 1).

Digital phenotyping data are prone to missingness. Therefore, we developed 2 measures to identify whether data had been successfully collected by Behapp for each hour, with one measure reflecting overall data availability and the other reflecting data availability specific to GPS (a sensor that is especially prone to missingness). These measures were required so that we could differentiate between values that were 0 because a participant was not using their phone and values that were 0 because data were not successfully collected. These measures capitalized on the sampling frequency of the location and other data sources such as Wi-Fi data (which are both independent of active phone usage). This frequency was expected to be greater than once per hour. A frequency <1 sample per hour in the location data indicated missing location data, and a frequency <1 sample per hour in all types of data (including Wi-Fi) indicated that overall data were not being collected successfully. Therefore, one of these measures reflected overall data availability, and the other measure was specific to GPS data availability. The distributions for these measures are provided in Figures S2-S5 in Multimedia Appendix 1. Due to low GPS data availability acquired using the version of Behapp used in these studies, it was decided not to include the GPS channels in this analysis. Therefore, any missingness that occurred in the included channels occurred across all channels at the same timepoints (ie, it is not possible to have data missing at a time point in, for example, only the social media channel and not the other channels).

To account for any changes in behavior that may have arisen from study onboarding (ie, participant attending assessments at the study location), the first day of each participant’s Behapp data were excluded. Consequently, all time series began at midnight. If the overall data availability measure indicated missing data, then the channels were marked as “NA.” Since missing data are handled natively by the HMM implementation we used [22], as explained subsequently, no missing data imputation was carried out on the data.

#### Division Into Training and Validation Sets Based on Missing Data and Diagnostic Group

Participants were split into training and validation sets, with the training set used to train the model and the validation set used to investigate relationships between HMM-derived digital phenotypes and clinical measures. All participants with schizophrenia, AD, or SCC were assigned to the validation set (as well as a subset of withheld HCs), so that the HMM could be trained on HCs, akin to training on a reference category [10]. To ensure that the HMM was trained on high-quality data (ie, time series with low levels of missingness), HCs meeting an overall data availability criterion of at least 90% of timepoints available across their time series were assigned to the training set. No minimum requirement was set for Behapp participation length, so shorter time series that did not have missingness issues during data collection were still included. We randomly selected 15 of these high data availability HCs and retained them in the validation set, to allow for some amount of data availability matching between HCs in the training and validation sets, also increasing the number of HCs in the validation set with social and clinical scale measures available. The distributions of time series lengths for training and validation participants are provided in Figures S6 and S7 in Multimedia Appendix 1, and distributions of data availability are provided in Figures S2-S5 in Multimedia Appendix 1. In addition, we investigated equivalent HMMs trained on the entire dataset (ie, no training or validation split) for insight into how the dataset split was affecting the learned model.

### Overview of HMM

**Figure 1:**
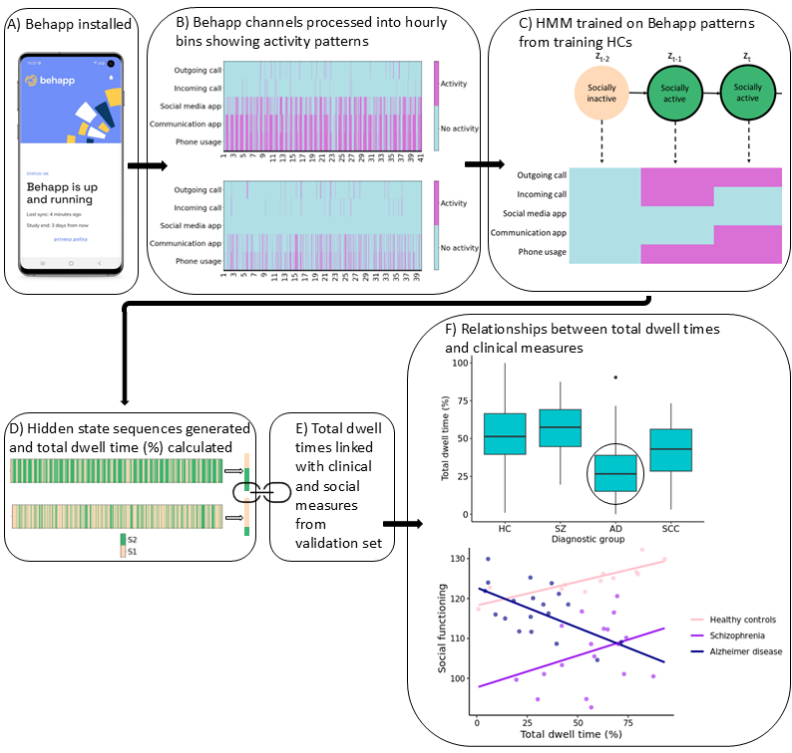
Overview of the hidden Markov model (HMM) approach showing the main processing and modeling steps involved in the method. (A) The Behapp app was installed and collected data passively. (B) These data were processed into activity bins. (C) The HMM was trained on the binned hourly time series. (D) The hidden state sequence was generated for each validation participant and their total dwell time calculated. (E) The total dwell time was compared to clinical measures. (F) Lower socially active dwell time in AD versus HCs, and an interaction between socially active dwell time and AD when predicting social functioning, were observed. AD: Alzheimer disease; HC: healthy control; SCC: subjective cognitive complaints; SZ: schizophrenia; S1: state 1; S2: state 2; z_t_: hidden state at time point, t.

An overview of the main approach used in this study can be seen in Figure 1. The HMM was used to model the observed smartphone data channels using a smaller number of hidden states, where each hidden state has corresponding probable values in these observed channels. Through time, the participant then switched between different hidden states. Mathematically, given a sequence of observed variables x_t_ and hidden states z_t_, at time t=1,...,T, the joint distribution for this model can be specified as follows:

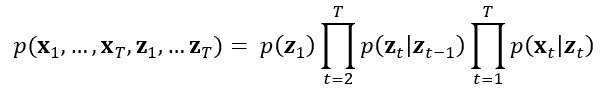



Where we use “1-hot” encoding for the latent variable, such that z_tn_=1 if the latent variable at time t belongs to the class n, and 0 otherwise. The different components of this model are described in greater detail in the subsequent sections.

The HMM model was implemented and fitted using the R package *depmixS4* [22]. During model training, the expectation-maximization algorithm was used to maximize the expected joint log-likelihood of the model parameters. The *depmixS4* package allows for missing values in the dataset, which means that missing values are effectively omitted from the calculation of the log-likelihood, and allows the specification of time-varying covariates that influence the transition probabilities as we outline subsequently. Although *depmixS4* allows for covariates to be specified over the starting probabilities, we did not explore this here. Each response variable (ie, observed channel) was modeled using a multinomial distribution with an identity link function. As all the input channels were binned into binary bins to manage the zero inflation, this resulted in a binomial distribution for each response variable.

We investigated a range of the number of hidden states used by the HMM. As the input data included a total of 5 channels, a reasonable number of hidden states used by the HMM to achieve data compression ranged from 2 to 4 states. Due to this small range of number of hidden states, this hyperparameter was not formally optimized, but rather we selected 1 main model for reporting and reported results from the additional relevant models in Multimedia Appendix 2. We also reported the Bayesian information criteria (BIC) for the various models. In addition, we investigated the inclusion of the time of day (ie, the hour) as a covariate in the model (ie, over the transition probabilities) and used the BIC to determine whether to include this covariate in the models used for subsequent analyses. As the hour is recorded as ranging from 0 (midnight) to 23 (11 PM), the hour must be encoded so that it is not incorrectly implied that, for example, midnight is distant from 11 PM. Therefore, we used 1-hot encoding to encode the hour (where an indicator variable is used for each hour). We also investigated different seeds for model training; however, this did not impact the likelihood of the model.

We then applied the trained HMM to the validation dataset and generated the hidden state sequences corresponding to these participants’ time series using the Viterbi algorithm. Note that this step did not involve retraining the model, and that the hidden state sequence was equal in length to the observed time series. For the alternative HMMs trained on the whole dataset, the hidden state sequences were generated for all participants, and subsequent investigations were made for all participants.

### HMM Parameters and Measures

Various probabilities reflecting each of the hidden states were learned during model training, which can be used to describe the model and to understand what behaviors each of the hidden states is associated with. This includes emission, starting, and transition probabilities.

#### Emission Probability

The emission probability for each state refers to the probability that certain values in each of the observed channels are observed given that the sequence is in that hidden state and can, therefore, be used to interpret what observed behaviors each hidden state represents. A state may give a high probability of observing activity in some observed behavioral channels and not others, and this can be identified with the emission probability. The emission probabilities of observed values x_t_ at time t given hidden states z_t_ are given by:

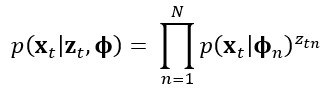



Where ϕ is a set of parameters governing the distribution of the observed data, N is the total number of hidden states in the model, that is, in our case, ranging from 2 to 4 for the different HMMs investigated.

#### Starting Probability

The starting probability indicates the probability of beginning the sequence in each hidden state. If a time series often begins with the same observed values, then the hidden state corresponding to these values will have a high starting probability. The probability distribution gives the probability that each hidden state will be the first hidden state, z_1_ is given by:

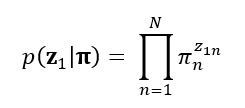



Where π is the probability vector with elements π_n_ ≡ p(z_1n_ = 1).

#### Transition Probability

The transition probability gives the probability of switching into another hidden state from each state (or the probability of staying in the same state). For example, for behaviors with long durations, the transition probability of staying in the associated hidden state may be high relative to the probability of transitioning to a nonrelated hidden state. The probability of transitioning into each hidden state at time t is dependent on the previous hidden state, and is given by:

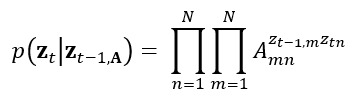



Where the elements of A are each of the transition probabilities such that A_mn_ ≡ p(z_tn_ = 1|z_t-1,m_ = 1, c_t_) denotes the probability of transitioning from state m to state n at time t and we make it explicit that this can depend on a vector of time-varying covariates c_t_.

In addition, other measures can be calculated from the hidden state sequence itself. In this study, we focused on a measure referred to as the “dwell time.”

#### Dwell Time

The dwell time per hidden state, also known as fractional occupancy, gives the percentage of time during which a state was occupied. This can be calculated for any desired level of granularity, for example, for all participants together, for each participant, for a specific period, or for each instance a state is occupied. In this study, we chose to calculate the *total* dwell time per participant, that is, a single dwell time value per participant in the validation set reflecting the percentage of their time series that was spent in the socially active state. We chose this level of granularity as we had a single value from each social functioning and clinical measure available per participant, that is, no repeated measures were available. As the validation set contained a range of data availability, any missing data timepoints were dropped from the time series after hidden state sequence generation, so that the calculation of total dwell time only reflected the available data. As we focused on a 2-state model in this study, we concentrated solely on the total dwell time spent in 1 state (the “socially active” state) and do not refer to the total dwell time of the other state in the analyses. For HMMs with more hidden states reported in Multimedia Appendix 2, we provide results for the states identified as socially active (refer to Figures S1-S10 in Multimedia Appendix 2 for the emission probabilities used to interpret each of the hidden states from the alternative models and their corresponding transition probabilities).

### Generalizability

As our principal model involved training on HCs, this could mean that the model was biased toward this population and not necessarily appropriate to use in other populations. To investigate whether a model trained on HCs can generalize sufficiently to the diagnostic groups, we investigated 2 additional models—a model trained on all the HCs and a model trained on all the remaining groups. We focused on 2-state models here and included the hour as a covariate over the transition probabilities following the same procedure as earlier. We compared the emission probabilities of these 2 models to establish whether equivalent hidden states were learnt and then generated hidden state sequences for the participants in the diagnostic groups using both models. We then compared these hidden state sequences by evaluating the accuracy, sensitivity, and specificity of the hidden state sequences provided by the HC model relative to the sequences provided by the diagnostic group model.

### Comparison of Total Dwell Time to Social and Clinical Measures

The total dwell times were used to predict 2 social measures using linear regression models—social functioning (SFS) [36] and loneliness [37] (available for participants in the PRISM study). For each of these measures, total dwell time, age, diagnostic group, and interactions between diagnostic group and total dwell time were included as predictors. For the SFS, separate models were also run for each of the diagnostic groups, with age included as an additional predictor.

Total dwell times were then compared between the different diagnostic groups and HCs (available for participants in both PRISM and Hersenonderzoek studies) using multinomial logistic regression, with total dwell time and age included as predictors. Sensitivity analyses of age were also carried out for each diagnostic group due to the broad age range in HCs because of age-matching to both the schizophrenia and AD groups and expected possible generational differences in phone use. For the schizophrenia sensitivity analysis, the maximum age for participants with schizophrenia was used as the maximum cut-off age for HCs (so age-matched HCs for the schizophrenia age sensitivity analysis had a maximum age of 41 years). For AD and SCC groups, each respective minimum participant age was used as the minimum cutoff age for HCs (so age-matched HCs for the AD sensitivity analysis had a minimum age of 51 years, and for the SCC sensitivity analysis, a minimum age of 44 years). Binomial logistic regression models were then run for each diagnostic group compared to their respective improved age-matched HCs.

Linear regression models were also run to predict cognitive impairment (MMSE; available for the participants with AD and HCs in the PRISM study) and schizophrenia symptoms (PANSS; available for the participants with schizophrenia in the PRISM study) from total dwell time. For MMSE, total dwell time, age, diagnostic group, and interactions between diagnostic group and total dwell time were included as predictors. In the case of PANSS scores, separate models were run to predict the total score and the subscores (positive, negative, general psychopathology, and composite) from total dwell time and age.

To assist readability, we present the results from total dwell times from a single HMM in this paper. The equivalent results from additional HMMs can be found in Multimedia Appendix 2.

## Results

### Sample Statistics

This study used data from participants in the PRISM and Hersenonderzoek datasets, which jointly contained 71% (247/348) HCs, 5.2% (18/348) participants with schizophrenia, 7.5% (26/348) participants with AD, and 16.4% (57/348) participants with SCC (Table 1). Participants with AD and HCs were present in both datasets, whereas participants with schizophrenia were provided by PRISM, and participants with SCC were provided by Hersenonderzoek.

**Table 1:** Demographics of each of the diagnostic groups.

Diagnostic group	Age (y), mean (SD)	Sex, n (%)			Dataset, n (%)			Country, n (%)			Education (y), mean (SD)
		Female	Male	PRISM^a^		HO^b^	NL^c^		ES^d^		
Healthy control (n=247)	59 (13)	140 (57)	107 (43)	28 (11)		219 (89)	234 (95)		13 (5)	6 (4)	
Schizophrenia (n=18)	31 (6)	7 (39)	11 (61)	18 (100)		0 (0)	12 (67)		6 (33)	15 (3)	
Alzheimer disease (n=26)	67 (7)	10 (38)	16 (62)	19 (73)		7 (27)	18 (69)		8 (31)	13 (7)	
Subjective cognitive complaints (n=57)	61 (7)	36 (63)	21 (37)	0 (0)		57 (100)	57 (100)		0 (0)	5 (2)	

^a^PRISM: Psychiatric Ratings using Intermediate Stratified Markers.

^b^HO: Hersenonderzoek.

^c^NL: the Netherlands.

^d^ES: Spain.

**Figure 2:**
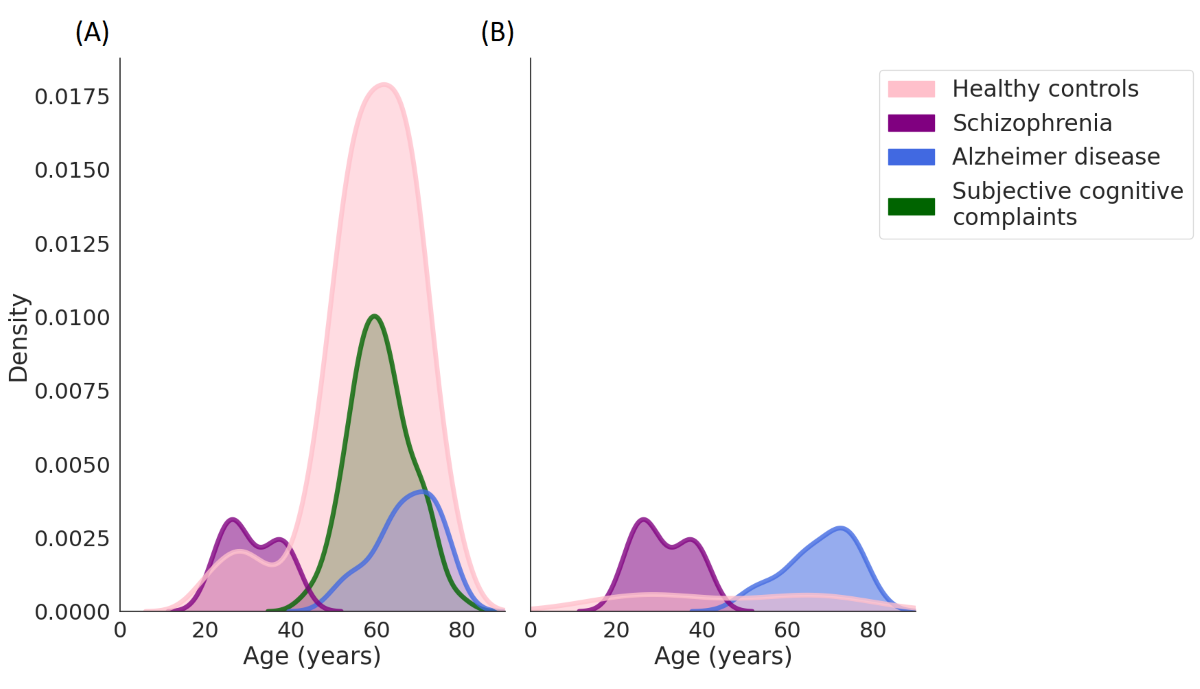
Age density distributions for validation participants. (A) Distribution of ages for all validation participants. (B) Distribution of ages for validation participants with social measures. Plotted using kernel density estimation.

In the PRISM and Hersenonderzoek datasets, HCs were age matched to the diagnostic groups, with the PRISM sample being matched to both participants with schizophrenia and AD and the Hersenonderzoek sample age-matched only to participants with AD. After aggregation of datasets, this resulted in a bimodal age distribution. Specifically, due to the expected differences in age between participants with schizophrenia and AD, the HCs were on average older than participants with schizophrenia and younger than those with AD. However, it is to be noted that the difference in age between the diagnostic groups is a consequence of aggregating multiple samples. From the age distributions presented in Figure 2, it is clear that the HC group spans the full range of each diagnostic group. We also performed additional sensitivity analyses with HCs age-matched to the diagnostic groups to confirm group comparison findings. Training set and overall validation set age distributions are shown in Figure S8 in Multimedia Appendix 1.

PRISM data were collected across sites in the Netherlands and Spain, while Hersenonderzoek data were collected solely in the Netherlands. PRISM recorded participant race, with nearly all participants identifying themselves as White, whereas Hersenonderzoek did not report participant race. The demographics of the HCs, split by training versus validation set assignment, are provided in Table S1 in Multimedia Appendix 1.

### HMM Derivation and Interpretation

When training the HMM, the number of hidden states used by the model must be set. We evaluated 2-, 3-, and 4-state models, which all converged. Generally, as the number of hidden states increased, the BIC improved, and it was also seen that including the hour as a covariate consistently improved the BIC (Table S1 in Multimedia Appendix 2). We have chosen to primarily present results from a 2-state model for simplicity, but we present equivalent results for other HMM variations in Multimedia Appendix 2. These alternative models varied in the number of hidden states (2-4) and the training set used (models trained on HCs with high data availability versus models trained on the entire dataset). For the models trained on all participants, total dwell times were also calculated for all participants (ie, not only the validation set).

**Figure 3:**
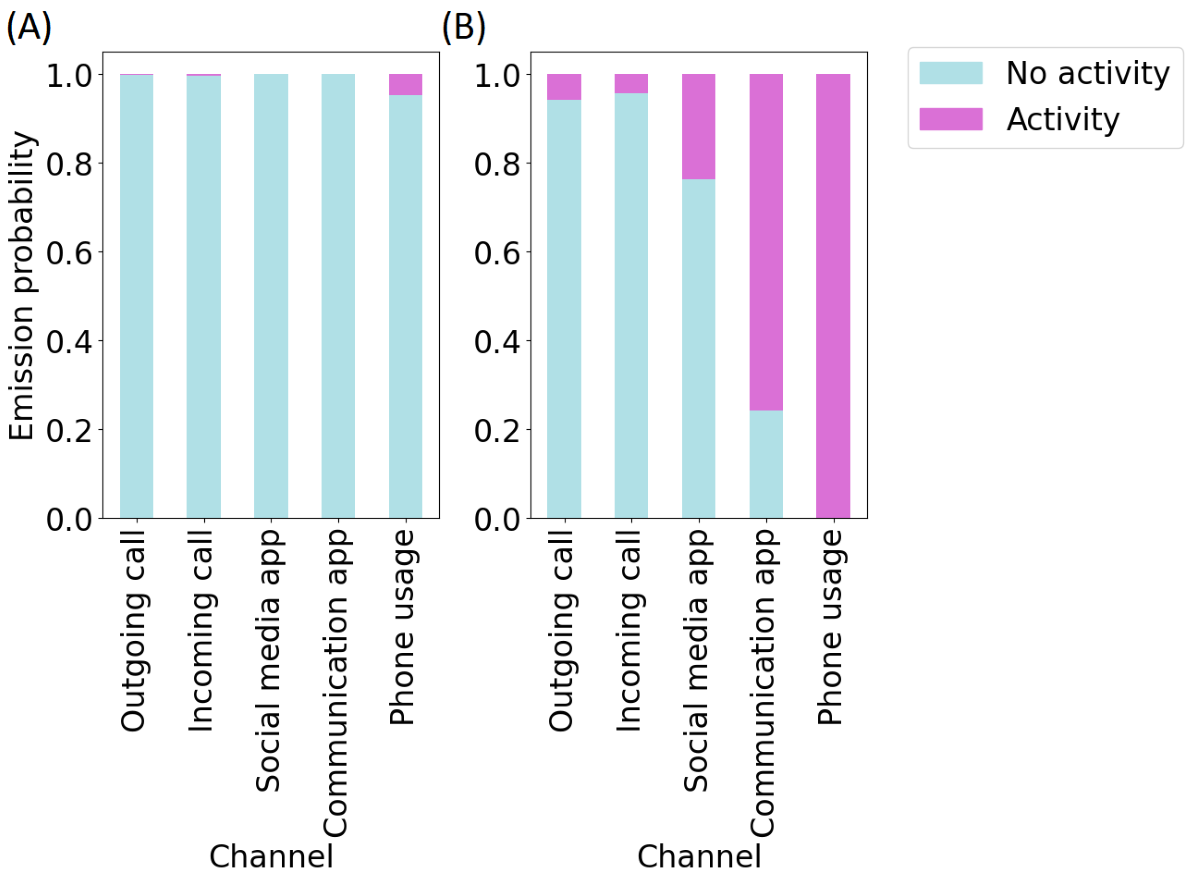
Emission probabilities of the selected 2-state model. Emission probabilities are provided for (A) state 1 (S1) and (B) state 2 (S2).

**Figure 4:**
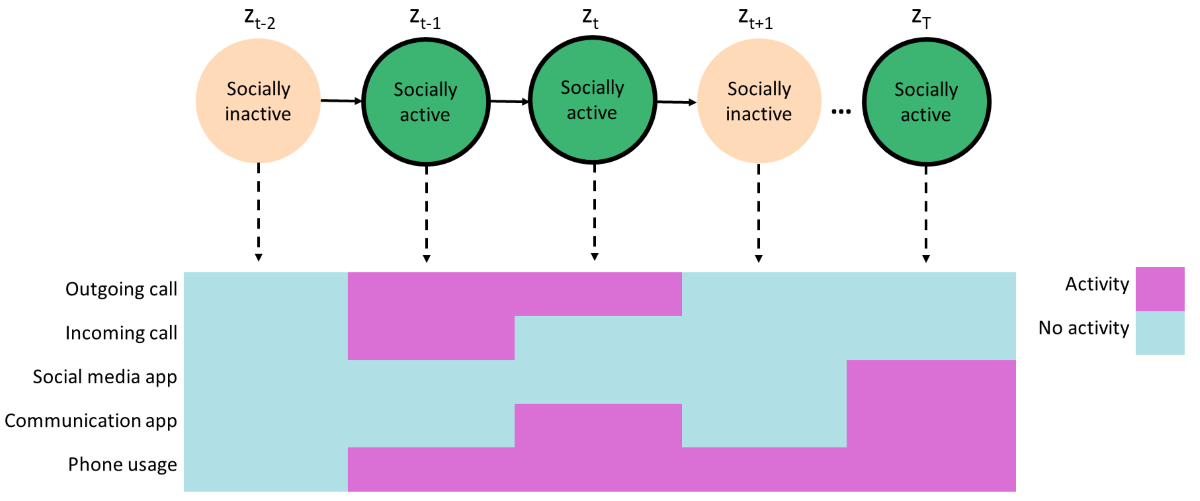
Examples of which behaviors may correspond to the hidden states. For the socially active state, various social behaviors are displayed, including calls and app use; in the socially inactive state, there may be no phone usage or phone usage without corresponding social behaviors.

The emission probabilities of the states generated by the 2-state model are shown in Figure 3. Using these emission probabilities to interpret the hidden states, it is evident that they represented socially active and socially inactive states. That is, the second state (S2) corresponded to phone usage with a very high probability that communication apps were also being used by the participant. There was a smaller probability of social media usage, and outgoing and incoming phone calls. Due to the use of communication methods in this state, such as calls and app usage, this hidden state was referred to as the “socially active” hidden state. The first state (S1) corresponded to a much smaller probability of phone usage, with the probability of all other channels near 0, and was referred to as the “socially inactive” hidden state. We show a demonstrative example of how the hidden states correspond to the observed channels in Figure 4, illustrating different observed channel configurations that can correspond to each of the hidden states.

After model training, the hidden state sequence corresponding to each participant’s time series was generated. The total dwell time for each validation participant could then be calculated from the hidden state sequence, with missing data in the validation set removed, and compared to clinical scores and diagnostic group. We chose to drop the missing portions from these time series after hidden state sequence generation due to the high rates of missingness for some participants. As the selected model only contained 2 states and the total dwell time (ie, the proportion of time spent in each state) was a percentage value, only the dwell times corresponding to 1 of the states needed to be investigated. Therefore, we focused on the total dwell times from the “socially active” state. Hence, further reference to “total dwell time” derived from the HMM solely refers to dwell times in the socially active state.

**Figure 5:**
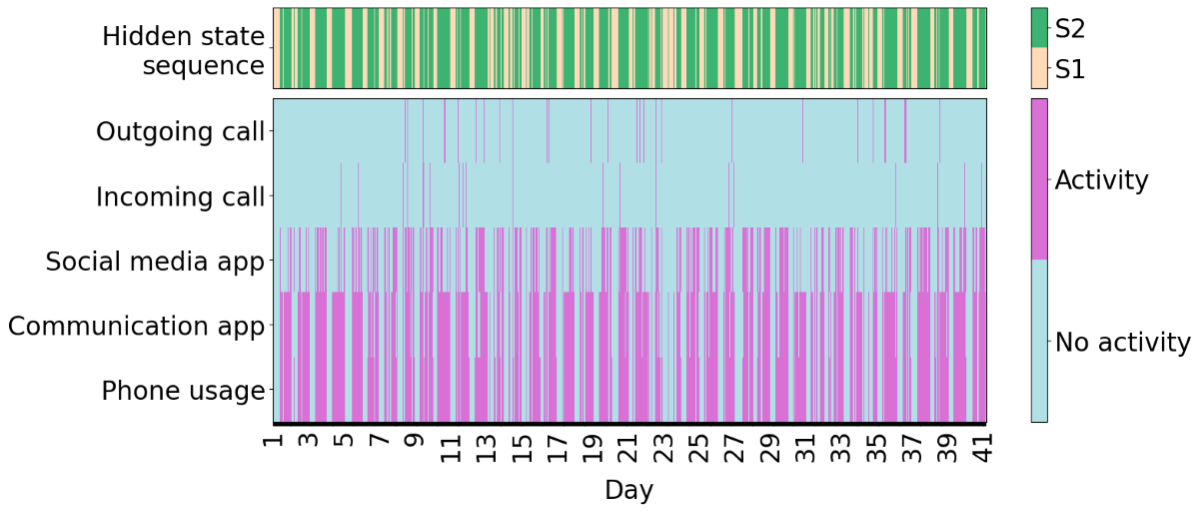
Example time series with high social activity. The observed time series composed of hourly bins (bottom 5 rows) of a participant compared with their corresponding predicted hidden state sequence (top row). S1: state 1 (socially inactive state); S2: state 2 (socially active state).

**Figure 6:**
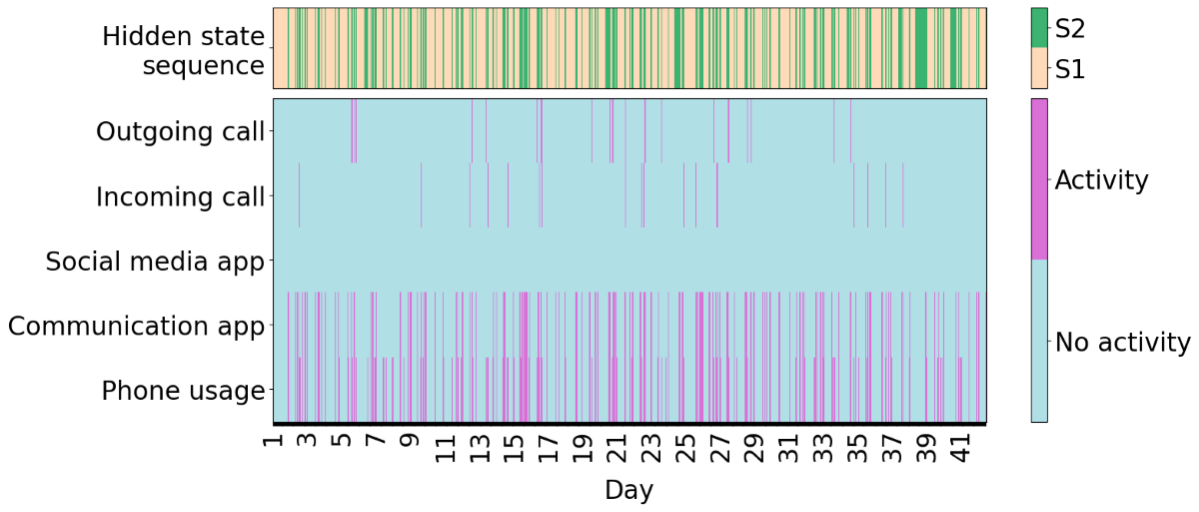
Example time series with low social activity. The observed time series composed of hourly bins (bottom 5 rows) of another participant compared with their corresponding predicted hidden state sequence (top row). S1: state 1 (socially inactive state); S2: state 2 (socially active state).

**Figure 7:**
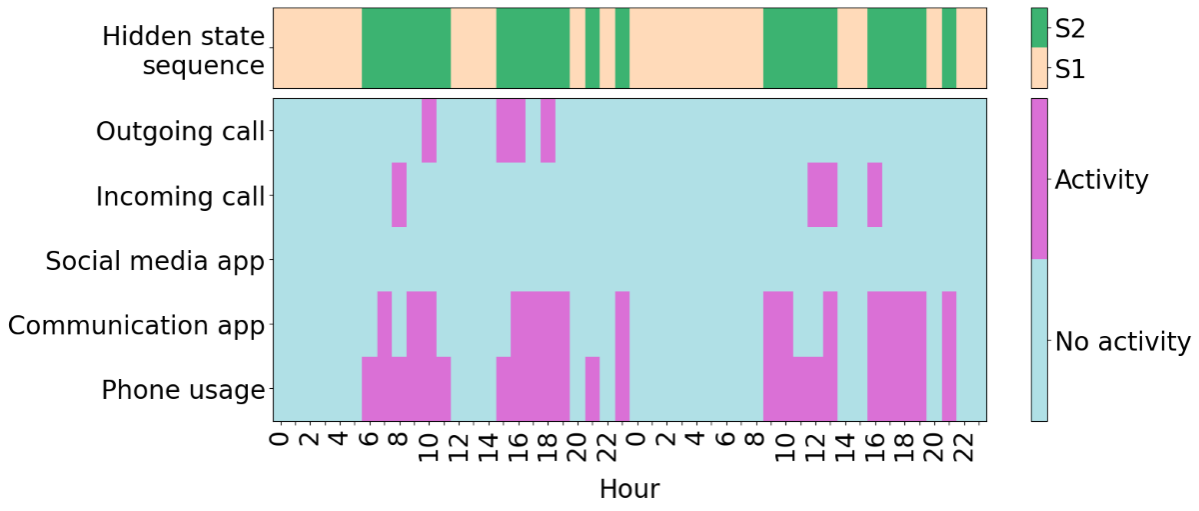
An example of a 2-day period of a participant’s time series. This participant showed higher social activity during the daytime than the nighttime. 0: midnight; S1: state 1 (socially inactive state); S2: state 2 (socially active state).

**Figure 8:**
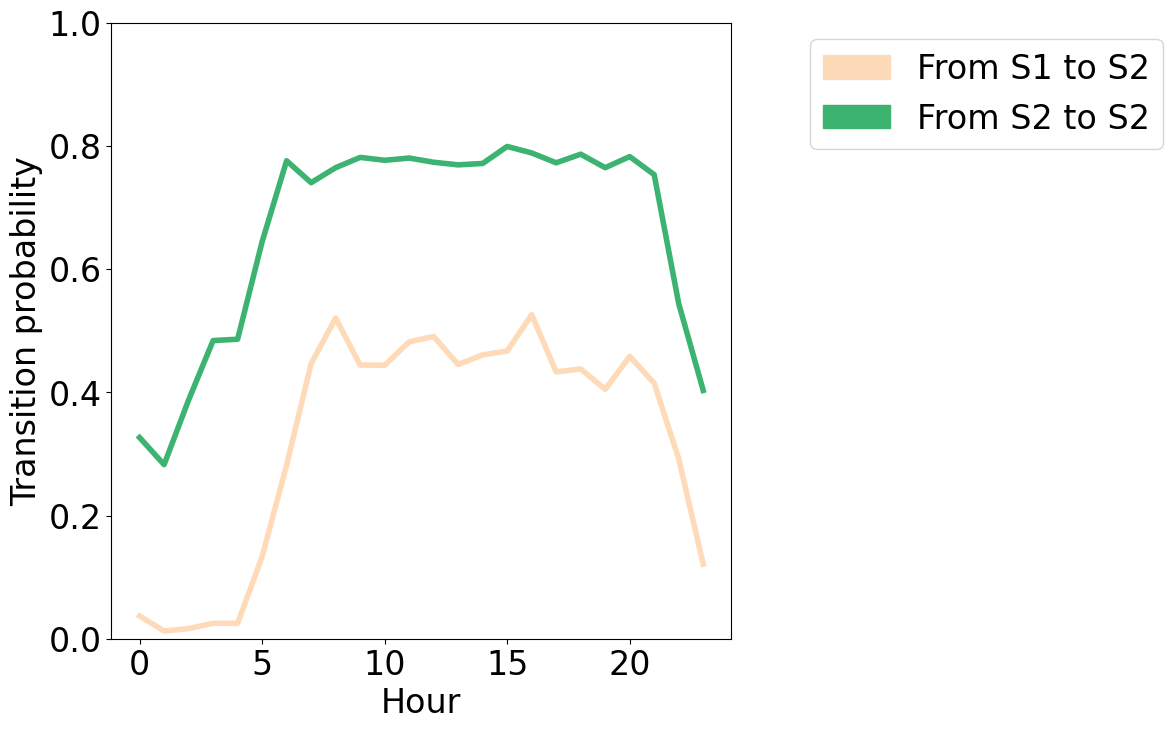
The probability of transitioning into the socially active state from each state, for each hour in the day. 0: midnight; S1: state 1 (socially inactive state), S2: state 2 (socially active state).

An example of one participant’s hidden state sequence alongside the input sequence is shown in Figure 5, and an example of another participant can be seen in Figure 6. It is immediately apparent that the participant shown in Figure 5 spends considerably more time in the socially active state relative to the participant shown in Figure 6. The participants in both Figures 5 and 6 oscillate quite frequently between the socially active and inactive states, which is not surprising due to expected diurnal variation [40]. More clearly, higher social activity during the daytime and lower social activity during the nighttime can be seen in Figure 7. In Figure 8, the probability of transitioning into the socially active state (state 2) from both the socially active and inactive states is increased during the daytime and drops off again in the evening. In addition, the probability of starting a hidden state sequence in the socially active and inactive states was 0.26 and 0.74, respectively, showing that it is more probable to begin the time series in the socially inactive state. This is to be expected, as all the time series began at midnight, so many participants would have been asleep.

### Generalizability

To investigate the generalizability of the approach of training the HMM using HCs and evaluating in other diagnostic groups, we compared the hidden state sequences of participants in the diagnostic groups generated from 2 different models—a model trained solely on these participants and a model trained solely on HCs. We found that both models produced very similar hidden states (Figures S11 and S12 in Multimedia Appendix 2), with state 1 in each model corresponding to social activity. Therefore, we did not need to relabel the hidden states before comparing the models. Specifically, considering the hidden state sequences from the model trained on all diagnostic groups as the “true” sequence, we found that the hidden state sequences from the model trained on HCs had an overall accuracy of 0.91, a sensitivity of being in the socially active state of 1.0, and specificity of 0.86. Overall, this suggests that an HMM trained on HCs can generalize adequately to the diagnostic groups in this analysis.

### Measures of Social Functioning and Loneliness

For validation purposes, we made use of a measure of social functioning for each participant in the PRISM dataset, namely the SFS [36] (see Figures S9 and S10 in Multimedia Appendix 1 for score distributions). Therefore, we investigated possible relationships between social functioning and total socially active dwell times for participants with SFS scores available. The number of participants in each group was small, so we considered our results to be preliminary indicators of possible relationships between the HMM-derived digital phenotypes and social functioning.

**Figure 9:**
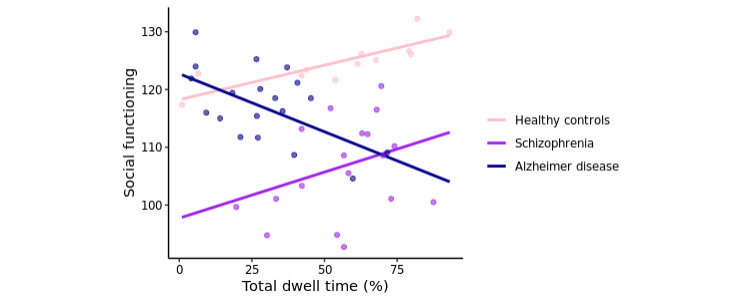
Social functioning scale score against total dwell time, with interactions displayed for the different groups.

To investigate the relationship between social functioning and total dwell time, we ran linear regression models that predicted SFS score from total dwell time, age, diagnostic group, and interactions between diagnostic group and total dwell time. HCs were taken as the reference group. False discovery rate (FDR)–corrected *P* values (considering 6 tests) were presented with results considered significant at *P*<.05 (Table 2). A significant interaction between the AD group and total dwell time was identified (FDR-corrected *P*=.02; Figure 9); however, no significant main effect of total dwell time was found. This result was robust across HMMs with different numbers of states and regardless of whether the model was trained on high–data availability HCs and assessed on withheld participants or trained and evaluated for all participants (Tables S2-S7 in Multimedia Appendix 2). In addition, a significant main effect of the schizophrenia group relative to HCs was seen (FDR-corrected *P*=.02), with lower SFS scores seen in the schizophrenia group, but no significant main effect of AD was seen.

**Table 2:** Results from a linear regression model predicting Social Functioning Scale score from total dwell time, age, and group, where healthy controls (12/49, 24%) were the reference group.

Predictor	Coefficient (SE)	*t* value (*df*=42)	*P* value	FDR^a^-corrected *P* value
Age	0.0269 (0.0788)	0.3406	.74	>.99
Schizophrenia group (n=18)	−20.5052 (6.6684)	−3.0750	.004	.02
Alzheimer disease group (n=19)	4.4857 (4.8877)	0.9178	.36	>.99
Total dwell time	0.1193 (0.0663)	1.7982	.08	.48
Interaction between the schizophrenia group and total dwell time	0.0401 (0.1069)	0.3757	.71	>.99
Interaction between the Alzheimer disease group and total dwell time	−0.3201 (0.1020)	−3.1384	.003	.02

^a^FDR: false discovery rate.

Linear regression models were also run within the different diagnostic groups to investigate possible within-group relationships between SFS scores and total dwell times. Separate models were run for each of the diagnostic groups in the validation set, with age included as an additional predictor in the models. FDR-corrected *P* values (considering 3 tests) are presented in Table S2 in Multimedia Appendix 1, with results considered significant at *P*<.05. A significant positive relationship between social functioning and total dwell times was found for the HCs (FDR-corrected *P*=.005), with every 1% increase in total dwell time corresponding to a 0.1153 increase in SFS score; however, this relationship was not seen when evaluating the entire HC group (using the HMM trained on all participants) and is expected to be due to sampling variation rather than differences in the learnt HMM parameters. No significant relationship was found for the other diagnostic groups.

A measure of loneliness [37] was also provided for the PRISM participants; however, no significant relationship between loneliness and total dwell times was found. The results from this linear regression model are presented in Table S3 in Multimedia Appendix 1, as well as histograms of the distribution of loneliness scores (Figures S11 and S12 in Multimedia Appendix 1).

### Diagnostic Group

**Figure 10:**
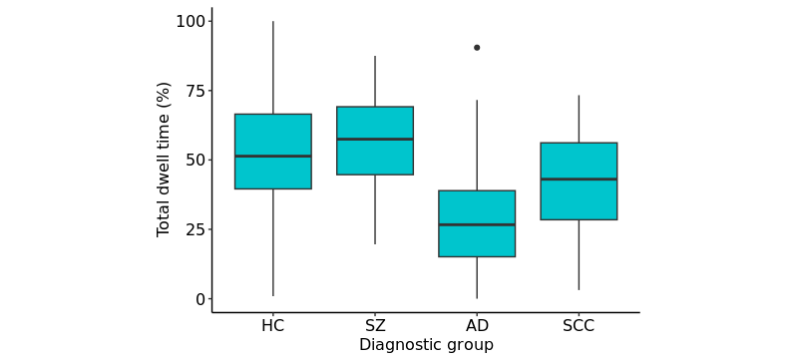
A box plot of the total dwell times per participant for the different diagnostic groups. There is a significant difference between the HC and AD groups, and the HC and SCC groups. AD: Alzheimer disease; HC: healthy control; SCC: subjective cognitive complaints; SZ: schizophrenia.

A multinomial logistic regression model was run to investigate differences in total socially active dwell time between the different diagnostic groups and the HC group in the validation set (ie, the reference category; Figure 10). Age was again included as an additional predictor in the model (age-related results are presented in Table S4 in Multimedia Appendix 1), and FDR-corrected *P* values (considering 3 tests) are presented to provide an indicator of significance at *P*<.05 (Table 3). Total dwell time was found to be a significant predictor of AD relative to HCs (FDR-corrected *P*<.001); participants with AD generally showed lower dwell times (ie, spending less time in the socially active state) relative to HCs (odds ratio 0.9483, 95% CI 0.9223-0.9742). This relationship was also seen across almost all equivalent socially active hidden states of the additional HMMs considered, with results of these models and their respective sensitivity analyses presented in Tables S8-S19 in Multimedia Appendix 2. For the SCC group, lower total dwell times were also observed relative to HCs (FDR-corrected *P*=.004, odds ratio 0.9742, 95% CI 0.9580-0.9903). However, this result was less robust when considering the other HMM variations. No significant relationship of total dwell time on the schizophrenia group was found relative to HCs. Due to the broad age range of HCs, sensitivity analyses of age were carried out for each diagnostic group (Table S5 in Multimedia Appendix 1), with a subsample of HCs age-matched to each respective diagnostic group, with the AD result remaining significant (FDR-corrected *P*<.001), along with the SCC result (FDR-corrected *P*=.003).

**Table 3:** Results from a multinomial logistic regression model predicting diagnostic group (vs healthy controls, 156/257, 60.7%) using total dwell time. Age was also included as a predictor.

Group	Coefficient (SE)	Odds ratio (95% CI)	*z* value	*P* value	FDR^a^-corrected *P* value
Schizophrenia (n=18)	−0.0133 (0.0172)	0.9867 (0.9531-1.0204)	−0.7763	.44	>.99
Alzheimer disease (n=26)	−0.0531 (0.0132)	0.9483 (0.9223-0.9742)	−4.0097	<.001	<.001
Subjective cognitive complaints (n=57)	−0.0262 (0.0082)	0.9742 (0.9580-0.9903)	−3.1846	.001	.004

^a^FDR: false discovery rate.

### Further Clinical Measures

For participants with AD and the HCs in the PRISM dataset, MMSE [35] scores, measuring cognitive impairment, were provided. No significant effect of total dwell time or age, nor a significant interaction between dwell time and diagnostic group, was found, although there was a significant effect of diagnostic group (Table S6 in Multimedia Appendix 1, with score distributions provided in Figures S13 and S14 in Multimedia Appendix 1). This was expected given the inclusion criteria of the study.

The PRISM dataset also provided PANSS [34] scores for participants with schizophrenia; however, no significant relationships between any of the PANSS scores (positive, negative, general psychopathology, composite, and total) and total dwell time were found. The results from these linear regression models are presented in Table S7 in Multimedia Appendix 1, as well as histograms of the distribution of PANSS scores per subscale (Figure S15 in Multimedia Appendix 1).

## Discussion

### Principal Findings

The central aim of digital phenotyping is to develop objective measures that can be used to monitor clinically relevant behaviors and symptom changes. In this study, we proposed a method for deriving meaningful, interpretable digital phenotypes using the HMM, a time series model that can accommodate missingness. We applied this model to general phone usage and communication smartphone measures, calculating the total socially active dwell time phenotyped by the HMM. Our smartphone measures were collected passively, reducing the burden on participants, and we protected participant privacy by abstracting app measures to descriptive levels, without collecting content. We investigated the association of the total socially active dwell time with various social and clinical measures, including diagnostic group and a questionnaire on social functioning (SFS). We found that 2- to 4-state HMMs provided comparable socially active states, which showed consistent results when investigating the relationships between the total dwell time and the social and clinical measures. We observed a significant difference in the HMM-derived total “socially active” dwell times between HCs and participants with AD, with participants with AD exhibiting lower total dwell times. This difference was robust to age sensitivity analysis and across different HMM variations (in terms of the number of hidden states and the training set used). A significant interaction between total dwell times and AD label was also observed for social functioning.

The HMM has several strengths. It uses lower-dimensional hidden states to represent the various observed behaviors, which can be easily interpreted for each state using the emission probabilities (Figure 3). The socially active state could be interpreted as being linked to observed communication-related behaviors, while the socially inactive state reflected a lack of these behaviors, such as other kinds of, or no, phone usage. Transitions between these hidden states indicated behavioral changes throughout time, for example, daily behavioral patterns (Figure 7). It was seen that during the daytime it was more likely for participants to transition to the socially active state than during the nighttime (Figure 8). Hidden states may allow for some individual behaviors to be represented as comparable behaviors. For example, Figure 6 shows a time series with no social media usage, whereas Figure 5 shows highly recurrent social media use, and both of these participants can have their respective behaviors represented using the socially active state despite individual differences in what social activity may mean for each participant. Therefore, this type of modeling approach can allow a certain amount of flexibility in the behaviors of the participants, dependent on the number of hidden states used in the model.

A summary measure of the HMM, the total socially active dwell time, was calculated per validation participant so that a model-derived digital phenotype could be compared to clinical and social measures. The observed difference in total dwell time between participants with AD and HCs, with participants with AD having lower dwell times than HCs, is consistent with the understanding that AD is associated with impaired social functioning [1] and demonstrates a potential objective measure of this difference. A significant interaction between total dwell time and AD was also seen when predicting social functioning, further demonstrating this.

Similarly to the AD group, differences in total dwell time relative to HCs were also observed for SCC participants; however, these differences were less robust across HMM variations. Differences in total dwell time were not observed for participants with schizophrenia. These results may be unsurprising as, by definition, participants with SCC are very similar to HCs, with the difference in inclusion criteria being that participants with SCC experience memory complaints. Similarly, the participants with schizophrenia did, for the most part, exhibit quite low symptom severity. The number of participants with schizophrenia was also small. While the PRISM study only placed exclusion criteria on positive symptoms (to exclude psychosis), the negative symptoms in the sample did not turn out to be very severe either, and overall, most participants could be classified as “mildly ill” based on their total PANSS score [41]. This indicates a selection of less-affected patients. The mild PANSS scores as well as low loneliness scores may also contribute to the absence of an identified relationship between these scales and total dwell time. When investigating social functioning within the different groups, significant relationships between social functioning and total dwell time for participants with AD and schizophrenia were also not observed. It is possible that participants with AD and schizophrenia may overestimate their social functioning [42], which could be reflected in their self-report SFS scores. This may complicate any possible relationship between this social functioning measure and total dwell time for these groups. A further interesting factor that could affect these relationships is the impact of different symptom profiles on total dwell time.

### Future Directions

To expand upon the current work, the HMM method could be applied in a larger population of participants with schizophrenia exhibiting broader symptom severity and different symptom profiles. Given the reluctance of many people with acute psychotic symptoms to being monitored, it may be necessary to monitor participants for a longer period, beginning with low symptom severity at study enrollment, to allow for more fluctuations in symptom severity to be observed [20]. The HMM method can also be applied to other disorders, including major depressive disorder (to be included in PRISM 2). A wider range of smartphone channels can also be included in the HMM, for example, calls could be encoded to reflect the variation in who is called and who is calling each hour. With a larger number of input channels, the derived hidden states could reflect more specific behavioral states. The optimal number of hidden states may then be driven by both the number of input channels and the underlying behavioral states of the participants themselves. With a higher-order model, the hidden states’ emission probabilities would not necessarily correspond to distinct single behaviors; for example, with the inclusion of GPS channels, there could be 2 hidden states that correspond to time spent at home, with one state also reflecting communication activities and the other reflecting no communication.

In our analysis, each hidden state sequence was generated per participant, but total dwell time comparisons were only made between groups. To shift toward individual predictions (for example predicting symptom scores or relapse along the time series), the dwell time for windows of the sequence or potentially the sequence likelihood could be extracted and changes along the time series evaluated. This would also maintain the time component of the analysis. Our current analysis uses a time series model but then compares a summary HMM measure to clinical measures. For clinical applications, the eventual goal would be to be able to make individual predictions along the time series. For this goal, it may be beneficial to include group-specific transition probabilities. It could also be beneficial to allow some individual parameters, for example, individualized transition probabilities could be considered. The choice of individual versus group-specific parameters may depend on the data available per individual. Zero-inflated distributions for the various channels could also be investigated as an alternative to binning the data into activity versus no activity bins.

Using our HMM trained on high data availability HCs as a reference group, we could identify differences in total socially active dwell time between the HCs and the AD group, as well as between HCs and the SCC group, and an interaction between the AD group and total dwell time on social functioning scores. These findings were also observed in models trained using the entire dataset. However, maintaining our dataset split in future analyses would allow us to look into the likelihood for identifying time series for which the model is a “poor fit,” that is, deviates from the reference group as a tool. This could be useful in datasets where, for example, the aim is to identify relapses, but for which there are not necessarily many examples of the relapse periods available that can be used in model training. These deviations could potentially be used to identify anomalous time series.

To improve the management of missing data, there are several more avenues that can be explored. Data are often expected to be missing due to technical difficulties, but it is also possible that data can be missing due to user behavior, for example, if the user switches the phone off, turns on flight mode, or deletes the app from their phone. It is also possible that a phone running out of battery could be correlated with certain activities carried out by the user or with certain times of the day, meaning the missingness is caused indirectly by user behavior. Future studies could consider recording the direct behaviors (which would currently be more feasible with Android phones, rather than iOS), to provide a better indicator of data missing due to technical difficulties versus user behavior.

### Limitations

Due to high rates of missingness, we made 4 main decisions to handle missing data: (1) to focus model training on high data availability time series, (2) to use a model that can accommodate missing data, (3) to exclude GPS channels from this time series analysis due to low levels of data availability affecting these specific channels, and (4) to exclude missing timepoints from the validation time series after hidden state sequence generation. While we view decisions (1) and (2) as useful strategies for managing missing data, decision (3), and to a lesser degree decision (4), were unfortunate consequences that in future studies should be avoided with improved data collection. The datasets used in this study were collected with early versions of Behapp, and throughout data collection, no indicator of missingness was known. Indicators of missing data were developed retrospectively using Wi-Fi and GPS sampling frequencies to assist analyses of these time series. Incoming data monitoring has now been improved in more recent Behapp versions, as well as the overall data collection process. Therefore, researchers using Behapp can now track data collection as it is ongoing and take action if sustained periods of data are missing. This could involve contacting participants to ensure they have not accidentally disabled desired functionalities for sustained periods. In addition, while the analysis package we used assumes that data are missing at random, and therefore, equally likely to be missing across the different hidden states, it is possible that this is not the case and that missingness may vary across hidden states. For example, in cases where missingness may be due to user behavior affecting battery consumption.

For interpretation purposes, we have named the 2 hidden states as “socially active” and “socially inactive.” However, a person could, of course, be socially active offline without using their phone. For example, a person may be socializing with friends at home without using their phone. Therefore, we acknowledge the limits to our naming convention and recommend caution when interpreting hidden states. Other sensors could be used to give an indicator of other people in the participant’s vicinity, such as Bluetooth [43], but passive smartphone data will nevertheless remain somewhat of a proxy for social activity. In a similar vein, we used the App Store classification to group apps, but participants may use the apps for purposes other than this classification (eg, some people use Instagram for communication, and less so for social media). While in our 2-state model these discrepancies would be inconsequential, with a larger number of hidden states, these discrepancies could potentially lead to misleading interpretations of a person’s behavior. In a clinical setting, the patient’s behaviors could be discussed with the clinician at the beginning of the Behapp use to assist in understanding and interpreting their personal digital phenotypes.

In addition, it is worth noting that by using a reference class approach, we do restrict the model to only learning hidden states present in the reference group (as well as transition and starting probabilities associated with the reference). While we also trained the HMM on all participants (Multimedia Appendix 2) and found that the hidden states present in our HMM trained on high data availability HCs were highly comparable to the hidden states in HMMs trained on the whole dataset, for other datasets (such as those with a larger number of input channels) it may be that those in a clinical group could exhibit different hidden states, or that if a clinical group in remission or with low symptom severity is used for model training that states associated with relapse or high symptom severity would not be learned by the model. Therefore, the dataset used for training the model and the subsequent analysis steps must be considered, as this restricts the hidden states that are learned by the model. In such cases, accepting that the trained model may not be a “good fit” for the withheld data could be something that could be used to help rather than hinder the analysis, by looking for deviations in the likelihood of such data with respect to the learned HMM.

### Conclusions

Smartphone-based digital phenotyping is a promising tool for monitoring and predicting mental health outcomes. However, methods are needed for managing this multifaceted time series smartphone data. We proposed the use of an HMM to model digital phenotyping time series, as this method can (1) combine different behavioral features, (2) reflect temporal behavioral changes, (3) be easily interpreted, and (4) manage missingness. We developed a 2-state model that represented various smartphone channels as “socially active” and “socially inactive” states, and calculated the total socially active dwell time for each participant’s time series. We identified a significant difference between HC and AD dwell times, with AD dwell times being lower than HC dwell times, showing how this HMM-derived digital phenotype may be a useful measure to indicate differences in social functioning. We also observed a significant interaction between total dwell time and the AD group when predicting social functioning. The HMM is an interpretable method to model behavior based on digital phenotyping data, and with further development, it can provide an appealing approach for making clinical predictions of symptom changes and relapse across a range of neuropsychiatric diseases.

## Appendix

Multimedia Appendix 1 Supporting results for the main hidden Markov model variation that is reported, as well as histograms reflecting different aspects of the Behapp data and participant scores.

Multimedia Appendix 2 Results from additional hidden Markov model (HMM) variations, including HMMs trained on the entire dataset and HMMs with 3-4 hidden states.

## Abbreviations

AD: Alzheimer disease
BIC: Bayesian information criteria
FDR: false discovery rate
HC: healthy control
HMM: hidden Markov model
MMSE: Mini-Mental State Examination
PANSS: Positive and Negative Syndrome Scale
PRISM: Psychiatric Ratings using Intermediate Stratified Markers
SCC: subjective cognitive complaints
SFS: Social Functioning Scale
