# Correction: Uncovering Social States in Healthy and Clinical Populations Using Digital Phenotyping and Hidden Markov Models: Observational Study

**DOI:** 10.2196/87810

**Published:** 2025-12-24

**Authors:** Imogen E Leaning, Andrea Costanzo, Raj Jagesar, Lianne M Reus, Pieter Jelle Visser, Martien J H Kas, Christian F Beckmann, Henricus G Ruhé, Andre F Marquand

**Affiliations:** 1 Donders Institute for Brain, Cognition and Behaviour Radboud University Nijmegen Nijmegen The Netherlands; 2 Department for Medical Neuroscience Radboud University Medical Center Nijmegen Nijmegen The Netherlands; 3 Groningen Institute for Evolutionary Life Sciences University of Groningen Groningen The Netherlands; 4 Department of Neurology, Alzheimer Center Amsterdam Neuroscience Amsterdam UMC Amsterdam The Netherlands; 5 Amsterdam Neuroscience Neurodegeneration Amsterdam UMC Amsterdam The Netherlands; 6 Center for Neurobehavioral Genetics, Semel Institute for Neuroscience and Human Behavior David Geffen School of Medicine University of California Los Angeles, CA United States; 7 Department of Psychiatry & Neuropsychology School for Mental Health and Neuroscience Maastricht University Maastricht The Netherlands; 8 Department of Neurobiology, Care Sciences and Society Division of Neurogeriatrics Karolinska Institutet Stockholm Sweden; 9 Department of Psychiatry Radboud University Medical Center Nijmegen Nijmegen The Netherlands; 10 Department of Neuroimaging Institute of Psychiatry, Psychology and Neuroscience King’s College London London United Kingdom

In “Uncovering Social States in Healthy and Clinical Populations Using Digital Phenotyping and Hidden Markov Models: Observational Study” [1] the authors noted one error.

The error pertains to the visualization of the transition probabilities in Figure 8, where a mistake in the calculation of these probabilities was made. This error did not impact any of the text in the paper as the interpretation of the figure remains the same, and the values were not used in any analysis. As the same code was used to visualize transition probabilities of alternative models in the supplementary materials, the transition probability figures in Multimedia Appendix 2 have also been corrected.

The correction will appear in the online version of the paper on the JMIR Publications website, together with the publication of this correction notice. Because this was made after submission to PubMed, PubMed Central, and other full-text repositories, the corrected article has also been resubmitted to those repositories.

**Figure 8:**
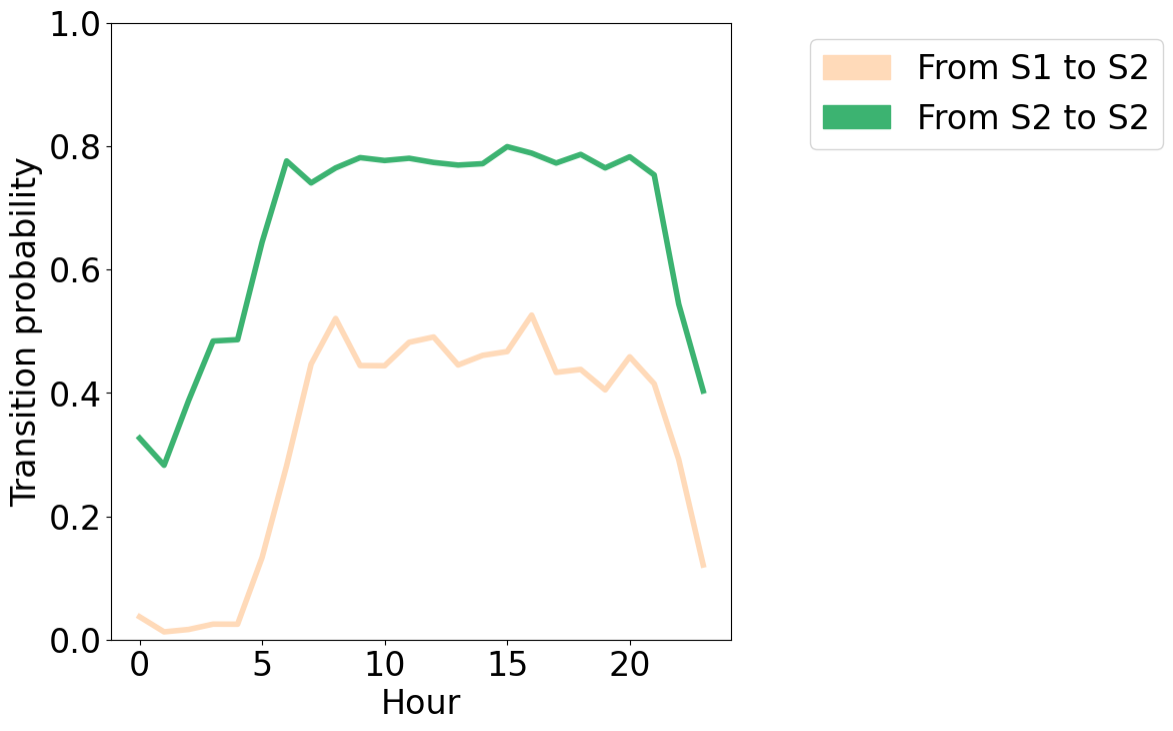
The probability of transitioning into the socially active state from each state, for each hour in the day. 0: midnight; S1: state 1 (socially inactive state), S2: state 2 (socially active state).

## Appendix

Multimedia Appendix 1 Results from additional hidden Markov model (HMM) variations, including HMMs trained on the entire dataset and HMMs with 3-4 hidden states.
