# Supplementary material for: Uncovering Social States in Healthy and Clinical Populations Using Digital Phenotyping and Hidden Markov Models: Observational Study
Source: J Med Internet Res. 2025 Apr 28;27:e64007. doi: 10.2196/64007 (PMC12070022; doi:10.2196/64007)
Supplement: Multimedia Appendix 2 [file jmir_v27i1e64007_app2.pdf]

Multimedia Appendix 2. Supplementary Materials

General information regarding additional HMMs

All of the relevant results for each HMM variation are reported one-by-one. For conciseness, we report the results from the state/s that were interpreted as socially active based on each HMM's emission probabilities, and where informative include a note of the results from statistical models from the other states.

Tables

Table S1: In-sample Bayesian Information Criteria (BIC) for the different HMM variations investigated.

Data used for training	Number of states	Hour included as covariate?	In-sample BIC
Training set	2	No	198580
Training set	3	No	195740
Training set	4	No	194315
Training set	2	Yes	188240
Training set	3	Yes	185295
Training set	4	Yes	185881
All	2	No	607748
All	3	No	594881
All	4	No	575001
All	2	Yes	586338
All	3	Yes	570134
All	4	Yes	548748

SFS

Table S2: Three state HMM trained using training set, including hour as a covariate - results from a linear regression model predicting SFS score from total dwell time (state 1), age and group, where withheld healthy controls (n=12) were the reference group.

Predictor	Coefficient	Standard error	*t* value	*P* value	FDR corrected *P* value
Age	0.0311	0.0809	0.3840	.70	1.0
Group: Schizophrenia (n=18)	-19.8194	6.2281	-3.1823	.003	.02
Group: Alzheimer's disease (n=19)	3.9336	4.6803	0.8405	.41	1.0
Total dwell time	0.1251	0.0692	1.8073	.08	.47
Interaction: Schizophrenia and total dwell time	0.0302	0.1057	0.2853	.78	1.0
Interaction: Alzheimer's disease and total dwell time	-0.3940	0.1248	-3.1572	.003	.02

No significant interaction between Alzheimer’s disease group and total state 2 dwell time was identified. A significant interaction between Alzheimer’s disease group and total state 3 dwell time was identified.

Table S3: Four state HMM trained using training set, including hour as a covariate - results from a linear regression model predicting SFS score from total dwell time (state 4), age and group, where withheld healthy controls (n=12) were the reference group.

Predictor	Coefficient	Standard error	*t* value	*P* value	FDR corrected *P* value
Age	0.0335	0.0828	0.4049	.69	.83
Group: Schizophrenia (n=18)	-18.7364	6.1746	-3.0344	.004	.01
Group: Alzheimer's disease (n=19)	3.4459	4.6335	0.7437	.46	.69
Total dwell time	0.1210	0.0676	1.7892	.08	.16
Interaction: Schizophrenia and total dwell time	0.0099	0.1018	0.0972	.92	.92
Interaction: Alzheimer's disease and total dwell time	-0.3781	0.1232	-3.0681	.004	.01

Table S4: Two state HMM trained using all data, including hour as a covariate - results from a linear regression model predicting SFS score from total dwell time (state 2), age and group, where all healthy controls (n=28) were the reference group.

Predictor	Coefficient	Standard error	*t* value	*P* value	FDR corrected *P* value
Age	0.0662	0.0542	1.2219	.23	1.0
Group: Schizophrenia (n=18)	-21.2049	5.8024	-3.6545	<.001	0.003
Group: Alzheimer's disease (n=19)	2.4022	3.8025	0.6318	.53	1.0
Total dwell time	0.0891	0.0530	1.6796	.10	0.59
Interaction: Schizophrenia and total dwell time	0.0734	0.0947	0.7751	.44	1.0
Interaction: Alzheimer's disease and total dwell time	-0.2813	0.0893	-3.1503	.003	0.02

Table S5: Three state HMM trained using all data, including hour as a covariate - results from a linear regression model predicting SFS score from total dwell time (state 1), age and group, where all healthy controls (n=28) were the reference group.

Predictor	Coefficient	Standard error	*t* value	*P* value	FDR corrected *P* value
Age	0.0650	0.0552	1.1777	.24	1.0
Group: Schizophrenia (n=18)	-22.7680	5.4685	-4.1635	<.001	<.001
Group: Alzheimer's disease (n=19)	1.9859	3.6657	0.5418	.59	1.0
Total dwell time	0.0830	0.0534	1.5538	.13	0.75
Interaction: Schizophrenia and total dwell time	0.1113	0.0951	1.1706	.25	1.0
Interaction: Alzheimer's disease and total dwell time	-0.3488	0.1109	-3.1444	.003	0.02

A significant interaction between Alzheimer’s disease and total state 2 dwell time was also identified. No significant interaction between Alzheimer’s disease and total state 3 dwell time was identified.

Table S6: Four state HMM trained using all data, including hour as a covariate - results from a linear regression model predicting SFS score from total dwell time (state 2), age and group, where all healthy controls (n=28) were the reference group.

Predictor	Coefficient	Standard error	*t* value	*P* value	FDR corrected *P* value
Age	0.0062	0.0481	0.1283	.90	.99
Group: Schizophrenia (n=18)	-9.0947	2.6567	-3.4234	.001	.003
Group: Alzheimer's disease (n=19)	0.0227	3.1312	0.0073	.99	.99
Total dwell time	0.0761	0.0449	1.6935	.10	.14
Interaction: Schizophrenia and total dwell time	-0.3583	0.0850	-4.2144	<.001	<.001
Interaction: Alzheimer's disease and total dwell time	-0.2499	0.0838	-2.9810	.004	.008

Table S7: Four state HMM trained using all data, including hour as a covariate - results from a linear regression model predicting SFS score from total dwell time (state 4), age and group, where all healthy controls (n=28) were the reference group.

Predictor	Coefficient	Standard error	*t* value	*P* value	FDR corrected *P* value
Age	0.0288	0.0612	0.4706	.64	.77
Group: Schizophrenia (n=18)	-24.4169	3.0941	-7.8914	<.001	<.001
Group: Alzheimer's disease (n=19)	-6.8078	2.0879	-3.2605	.002	.006
Total dwell time	-0.0023	0.0468	-0.0500	.96	.96
Interaction: Schizophrenia and total dwell time	0.2095	0.0697	3.0067	.004	.008
Interaction: Alzheimer's disease and total dwell time	-0.2527	0.2141	-1.1805	.24	.36

Diagnostic group

Table S8: Three state HMM trained using training set, including hour as a covariate - results from a multinomial logistic regression model predicting diagnostic group (versus withheld healthy controls (n=156)) using total dwell time (state 1).

Group	Predictor	Coefficient	Standard error	Odds	*z* value	*P* value	FDR corrected *P* value
Schizophrenia (n=18)	Total dwell time	-0.0115	0.0171	0.9886	-0.6711	.50	1.0
	Age	-0.1671	0.0306	0.8461	-5.4614	<.001	<.001
Alzheimer's disease (n=26)	Total dwell time	-0.0618	0.0148	0.9401	-4.1698	<.001	<.001
	Age	0.0789	0.0295	1.0821	2.6716	.008	.02
Subjective cognitive complaints (n=57)	Total dwell time	-0.0152	0.0084	0.9849	-1.8116	.07	.21
	Age	0.0152	0.0165	1.0153	0.9192	.36	1.0

The total state 2 dwell time was not a significant predictor of any of the diagnostic groups. Total state 3 dwell time was a significant predictor of Alzheimer's disease group and subjective cognitive complaints group.

Table S9: Three state HMM trained using training set, including hour as a covariate - Binomial logistic regression age sensitivity analysis results (for state 1). A model was run per diagnostic group, where the reference group for each model was age-matched healthy controls. Age cut-offs for healthy controls (HC) matched to each diagnostic group (schizophrenia (SZ), Alzheimer's disease (AD), subjective cognitive complaints (SCC)): HC vs SZ, Age < 42 (HC n=12); HC vs AD, Age > 50 (HC n=130); HC vs SCC, Age > 43 (HC n=142).

Group	Predictor	Coefficient	Standard error	Odds	*z* value	*P* value	FDR corrected *P* value
Schizophrenia (n=18)	Total dwell time	-0.0060	0.0194	0.9940	-0.3099	.76	1.0
	Age	0.0605	0.0638	1.0624	0.9488	.34	1.0
Alzheimer's disease (n=26)	Total dwell time	-0.0641	0.0156	0.9380	-4.1054	<.001	<.001
	Age	0.0518	0.0337	1.0531	1.5389	.12	.37
Subjective cognitive	Total dwell time	-0.0148	0.0083	0.9853	-1.7831	.07	.22

complaints (n=57)	Age	-0.0133	0.0207	0.9868	-0.6409	.52	1.0

Table S10: Four state HMM trained using training set, including hour as a covariate - results from a multinomial logistic regression model predicting diagnostic group (versus withheld healthy controls (n=156)) using total dwell time (state 4).

Group	Predictor	Coefficient	Standard error	Odds	*z* value	*P* value	FDR corrected *P* value
Schizophrenia (n=18)	Total dwell time	-0.0141	0.0166	0.9860	-0.8461	.40	1.0
	Age	-0.1687	0.0307	0.8448	-5.4909	<.001	<.001
Alzheimer's disease (n=26)	Total dwell time	-0.0579	0.0142	0.9437	-4.0775	<.001	<.001
	Age	0.0752	0.0297	1.0781	2.5295	.01	.03
Subjective cognitive complaints (n=57)	Total dwell time	-0.0186	0.0083	0.9815	-2.2462	.02	.07
	Age	0.0126	0.0166	1.0127	0.7617	.45	1.0

Table S11: Four state HMM trained using training set, including hour as a covariate - Binomial logistic regression age sensitivity analysis results (for state 4). A model was run per diagnostic group, where the reference group for each model was age-matched healthy controls. Age cut-offs for healthy controls (HC) matched to each diagnostic group (schizophrenia (SZ), Alzheimer's disease (AD), subjective cognitive complaints (SCC)): HC vs SZ, Age < 42 (HC n=12); HC vs AD, Age > 50 (HC n=130); HC vs SCC, Age > 43 (HC n=142).

Group	Predictor	Coefficient	Standard error	Odds	*z* value	*P* value	FDR corrected *P* value
Schizophrenia (n=18)	Total dwell time	-0.0054	0.0185	0.9946	-0.2915	.77	1.0
	Age	0.0602	0.0640	1.0621	0.9420	.35	1.0
Alzheimer's disease (n=26)	Total dwell time	-0.0587	0.0148	0.9430	-3.9704	<.001	<.001
	Age	0.0503	0.0334	1.0516	1.5077	.13	.39
Subjective cognitive complaints (n=57)	Total dwell time	-0.0192	0.0084	0.9810	-2.2887	.02	.07
	Age	-0.0176	0.0211	0.9826	-0.8359	.40	1.0

Table S12: Two state HMM trained using all data, including hour as a covariate - results from a multinomial logistic regression model predicting diagnostic group (versus withheld healthy controls (n=247)) using total dwell time (state 2).

Group	Predictor	Coefficient	Standard error	Odds	*z* value	*P* value	FDR corrected *P* value
Schizophrenia (n=18)	Total dwell time	-0.0182	0.0164	0.9820	-1.1087	.27	.80
	Age	-0.1490	0.0258	0.8615	-5.7663	<.001	<.001
Alzheimer's disease (n=26)	Total dwell time	-0.0553	0.0131	0.9462	-4.2351	<.001	<.001
	Age	0.0578	0.0266	1.0595	2.1678	.03	.09
Subjective cognitive complaints (n=57)	Total dwell time	-0.0260	0.0082	0.9744	-3.1518	.002	.005
	Age	0.0030	0.0141	1.0030	0.2162	.83	1.0

Table S13: Two state HMM trained using all data, including hour as a covariate - Binomial logistic regression age sensitivity analysis results (for state 2). A model was run per diagnostic group, where the reference group for each model was age-matched healthy controls. Age cut-offs for healthy controls (HC) matched to each diagnostic group (schizophrenia (SZ), Alzheimer's disease (AD), subjective cognitive complaints (SCC)): HC vs SZ, Age < 42 (HC n=24); HC vs AD, Age > 50 (HC n=206); HC vs SCC, Age > 43 (HC n=221).

Group	Predictor	Coefficient	Standard error	Odds	*z* value	*P* value	FDR corrected *P* value
Schizophrenia (n=18)	Total dwell time	-0.0126	0.0175	0.9875	-0.7160	.47	1.0
	Age	0.0423	0.0526	1.0432	0.8036	.42	1.0
Alzheimer's disease (n=26)	Total dwell time	-0.0557	0.0134	0.9458	-4.1505	<.001	<.001
	Age	0.0298	0.0303	1.0302	0.9810	.33	.98
Subjective cognitive complaints (n=57)	Total dwell time	-0.0283	0.0086	0.9721	-3.3007	<.001	.003
	Age	-0.0380	0.0196	0.9627	-1.9371	.05	.16

Table S14: Three state HMM trained using all data, including hour as a covariate - results from a multinomial logistic regression model predicting diagnostic group (versus withheld healthy controls (n=247)) using total dwell time (state 1).

Group	Predictor	Coefficient	Standard error	Odds	*z* value	*P* value	FDR corrected *P* value
Schizophrenia (n=18)	Total dwell time	-0.0211	0.0167	0.9791	-1.2642	.21	.62
	Age	-0.1529	0.0269	0.8582	-5.6748	<.001	<.001
Alzheimer's disease (n=26)	Total dwell time	-0.0647	0.0142	0.9373	-4.5734	<.001	<.001
	Age	0.0540	0.0269	1.0555	2.0117	.04	.13
Subjective cognitive complaints (n=57)	Total dwell time	-0.0187	0.0082	0.9815	-2.2625	.02	.07
	Age	0.0045	0.0144	1.0045	0.3098	.76	1.0

Total state 2 dwell time was a significant predictor of Alzheimer's disease group. However, total state 3 dwell time was not a significant predictor of any of the diagnostic groups.

Table S15: Three state HMM trained using all data, including hour as a covariate - Binomial logistic regression age sensitivity analysis results (for state 1). A model was run per diagnostic group, where the reference group for each model was age-matched healthy controls. Age cut-offs for healthy controls (HC) matched to each diagnostic group (schizophrenia (SZ), Alzheimer's disease (AD), subjective cognitive complaints (SCC)): HC vs SZ, Age < 42 (HC n=24); HC vs AD, Age > 50 (HC n=206); HC vs SCC, Age > 43 (HC n=221).

Group	Predictor	Coefficient	Standard error	Odds	*z* value	*P* value	FDR corrected *P* value
Schizophrenia (n=18)	Total dwell time	-0.0187	0.0180	0.9815	-1.0357	.30	.90
	Age	0.0386	0.0533	1.0394	0.7242	.47	1.0
Alzheimer's disease (n=26)	Total dwell time	-0.0663	0.0147	0.9358	-4.5234	<.001	<.001
	Age	0.0239	0.0312	1.0241	0.7644	.44	1.0
Subjective cognitive complaints (n=57)	Total dwell time	-0.0184	0.0082	0.9818	-2.2538	.02	.07
	Age	-0.0333	0.0195	0.9673	-1.7051	.09	.26

Table S16: Four state HMM trained using all data, including hour as a covariate - results from a multinomial logistic regression model predicting diagnostic group (versus withheld healthy controls (n=247)) using total dwell time (state 2).

Group	Predictor	Coefficient	Standard error	Odds	*z* value	*P* value	FDR corrected *P* value
Schizophrenia (n=18)	Total dwell time	0.0058	0.0134	1.0058	0.4362	.66	1.0
	Age	-0.1455	0.0262	0.8646	-5.5527	<.001	<.001
Alzheimer's disease (n=26)	Total dwell time	-0.0171	0.0114	0.9830	-1.5030	.13	.40
	Age	0.0897	0.0259	1.0939	3.4649	<.001	.002
Subjective cognitive complaints (n=57)	Total dwell time	-0.0152	0.0075	0.9849	-2.0275	.04	.13
	Age	0.0195	0.0135	1.0197	1.4428	.15	.45

Table S17: Four state HMM trained using all data, including hour as a covariate - Binomial logistic regression age sensitivity analysis results (for state 2). A model was run per diagnostic group, where the reference group for each model was age-matched healthy controls. Age cut-offs for healthy controls (HC) matched to each diagnostic group (schizophrenia (SZ), Alzheimer's disease (AD), subjective cognitive complaints (SCC)): HC vs SZ, Age < 42 (HC n=24); HC vs AD, Age > 50 (HC n=206); HC vs SCC, Age > 43 (HC n=221).

Group	Predictor	Coefficient	Standard error	Odds	*z* value	*P* value	FDR corrected *P* value
Schizophrenia (n=18)	Total dwell time	-0.0008	0.0145	0.9992	-0.0567	.95	1.0
	Age	0.0481	0.0537	1.0493	0.8958	.37	1.0
Alzheimer's disease (n=26)	Total dwell time	-0.0178	0.0114	0.9824	-1.5618	.12	.36
	Age	0.0650	0.0282	1.0671	2.3005	.02	.06
Subjective cognitive complaints (n=57)	Total dwell time	-0.0169	0.0075	0.9833	-2.2377	.03	.08
	Age	-0.0190	0.0188	0.9812	-1.0138	.31	.93

Table S18: Four state HMM trained using all data, including hour as a covariate - results from a multinomial logistic regression model predicting diagnostic group (versus withheld healthy controls (n=247)) using total dwell time (state 4).

Group	Predictor	Coefficient	Standard error	Odds	*z* value	*P* value	FDR corrected *P* value
Schizophrenia (n=18)	Total dwell time	-0.0157	0.0129	0.9845	-1.2144	.22	.67
	Age	-0.1556	0.0282	0.8559	-5.5164	<.001	<.001
Alzheimer's disease (n=26)	Total dwell time	-0.0817	0.0350	0.9216	-2.3359	.02	.06
	Age	0.0650	0.0277	1.0672	2.3454	.02	.06
Subjective cognitive complaints (n=57)	Total dwell time	-0.0059	0.0076	0.9941	-0.7717	.44	1.0
	Age	0.0114	0.0145	1.0115	0.7868	.43	1.0

Table S19: Four state HMM trained using all data, including hour as a covariate - Binomial logistic regression age sensitivity analysis results (for state 4). A model was run per diagnostic group, where the reference group for each model was age-matched healthy controls. Age cut-offs for healthy controls (HC) matched to each diagnostic group (schizophrenia (SZ), Alzheimer's disease (AD), subjective cognitive complaints (SCC)): HC vs SZ, Age < 42 (HC n=24); HC vs AD, Age > 50 (HC n=206); HC vs SCC, Age > 43 (HC n=221).

Group	Predictor	Coefficient	Standard error	Odds	*z* value	*P* value	FDR corrected *P* value
Schizophrenia (n=18)	Total dwell time	-0.0062	0.0126	0.9939	-0.4863	.63	1.0
	Age	0.0385	0.0549	1.0393	0.7017	.48	1.0
Alzheimer's disease (n=26)	Total dwell time	-0.0853	0.0361	0.9182	-2.3630	.02	.05
	Age	0.0429	0.0309	1.0439	1.3886	.16	.49
Subjective cognitive complaints (n=57)	Total dwell time	-0.0051	0.0077	0.9949	-0.6645	.51	1.0
	Age	-0.0252	0.0195	0.9751	-1.2930	.20	.59

Figures

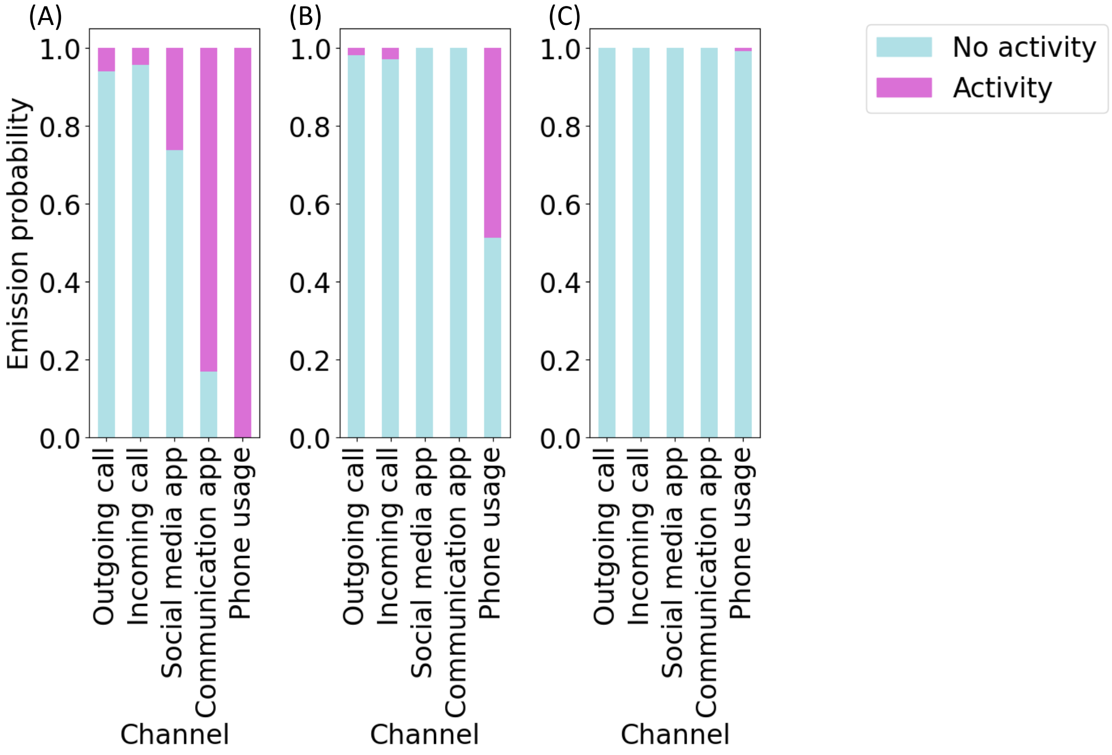

Figure S1: Emission probabilities for three state HMM trained using training set, including hour as a covariate, for (A) state 1, (B) state 2 and (C) state 3.

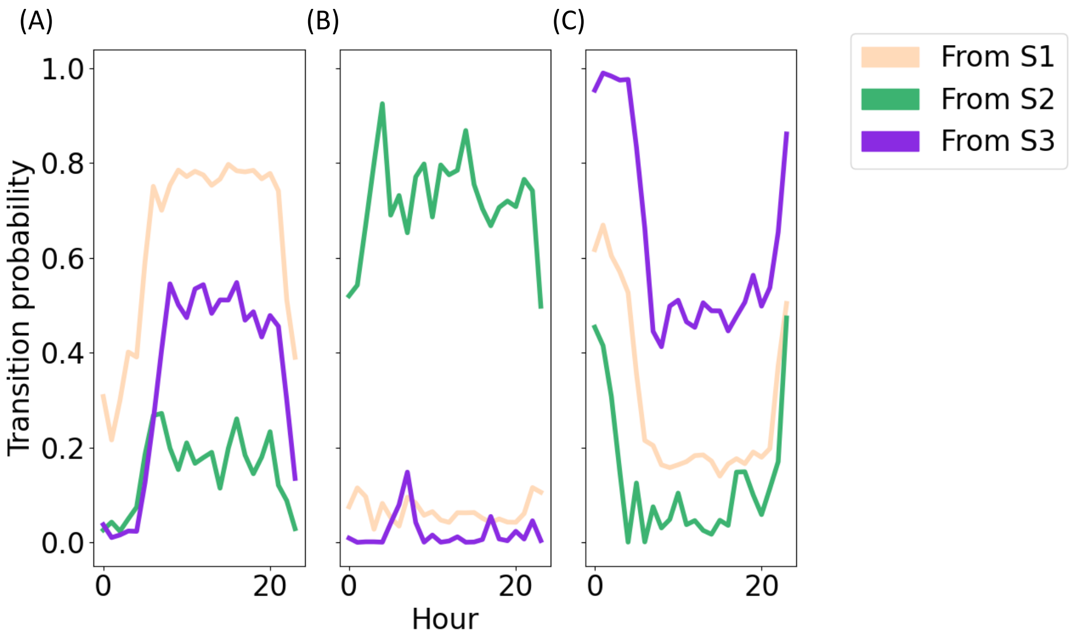

Figure S2: Transition probabilities for three state HMM trained using training set, including hour as a covariate, reflecting transitions to (A) state 1, (B) state 2 and (C) state 3.

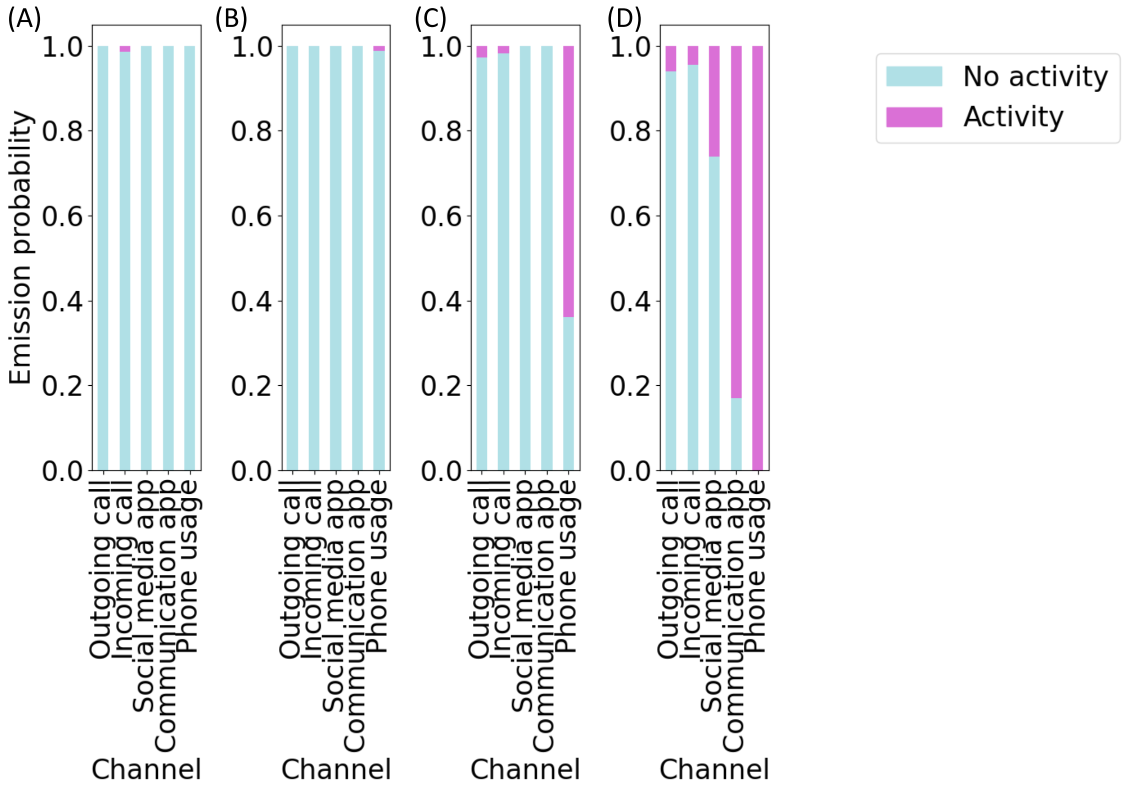

Figure S3: Emission probabilities for four state HMM trained using training set, including hour as a covariate, for (A) state 1, (B) state 2, (C) state 3 and (D) state 4.

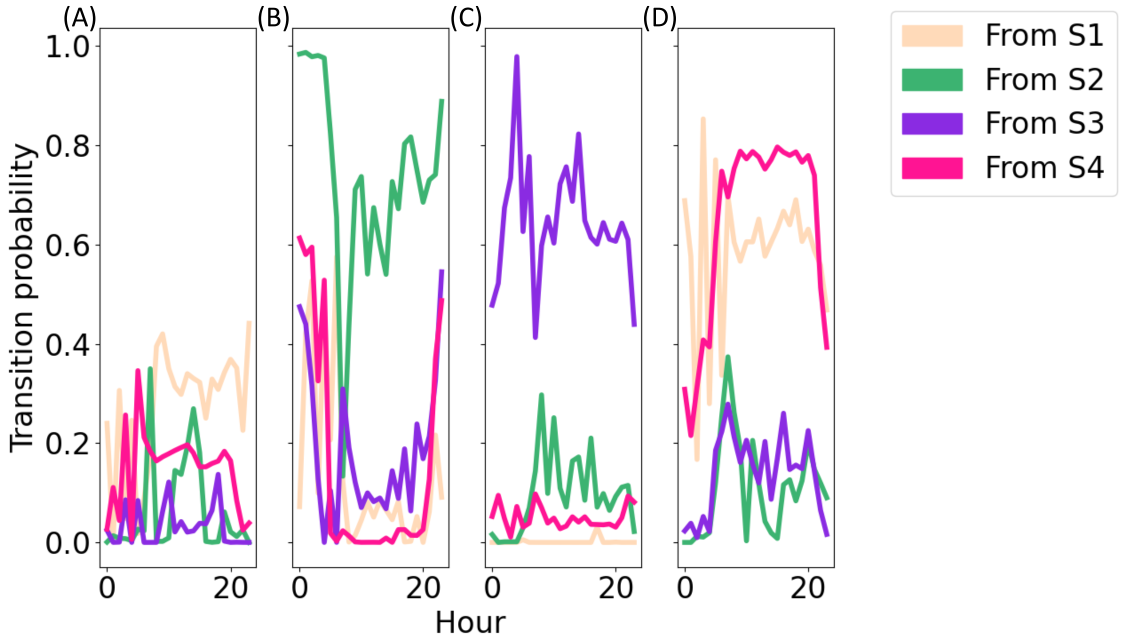

Figure S4: Transition probabilities for four state HMM trained using training set, including hour as a covariate, reflecting transitions to (A) state 1, (B) state 2, (C) state 3 and (D) state 4.

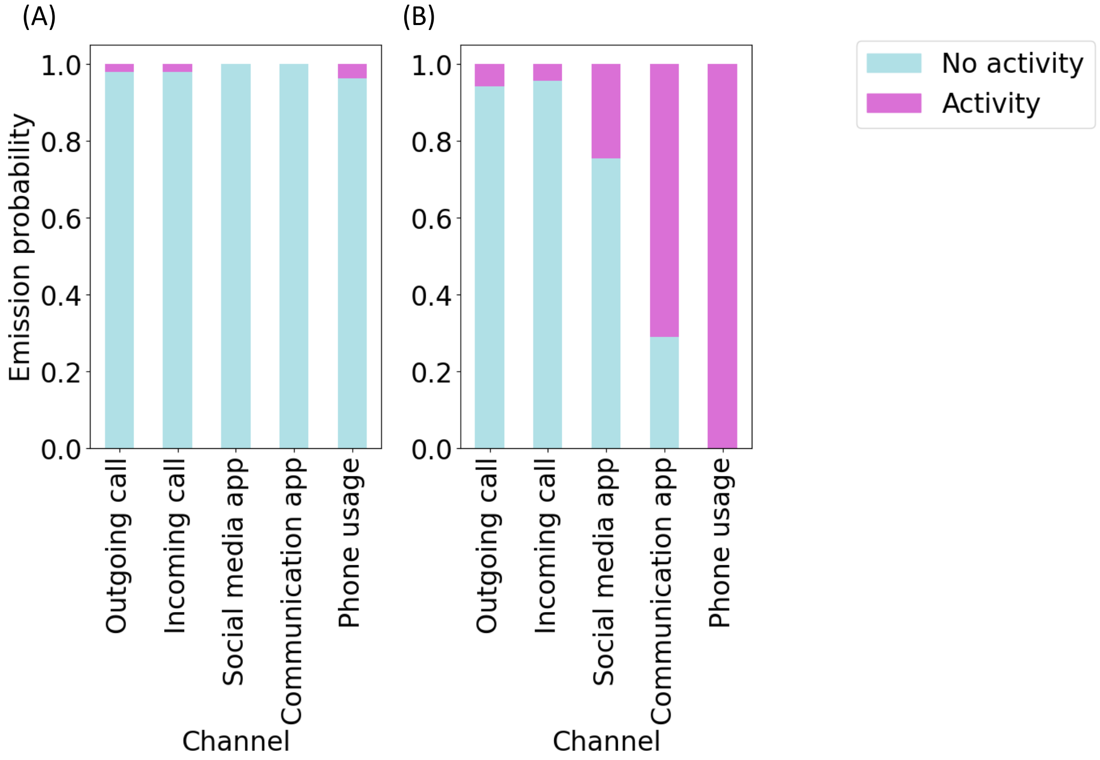

Figure S5: Emission probabilities for two state HMM trained using all data, including hour as a covariate, for (A) state 1 and (B) state 2.

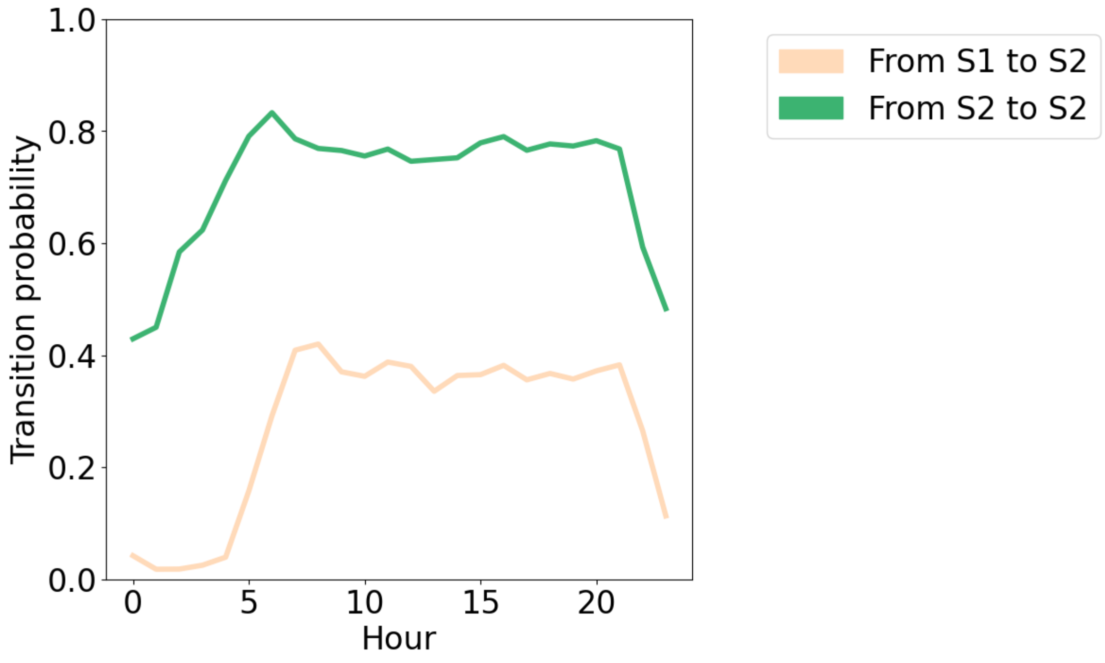

Figure S6: Transition probabilities for two state HMM trained using all data, including hour as a covariate.

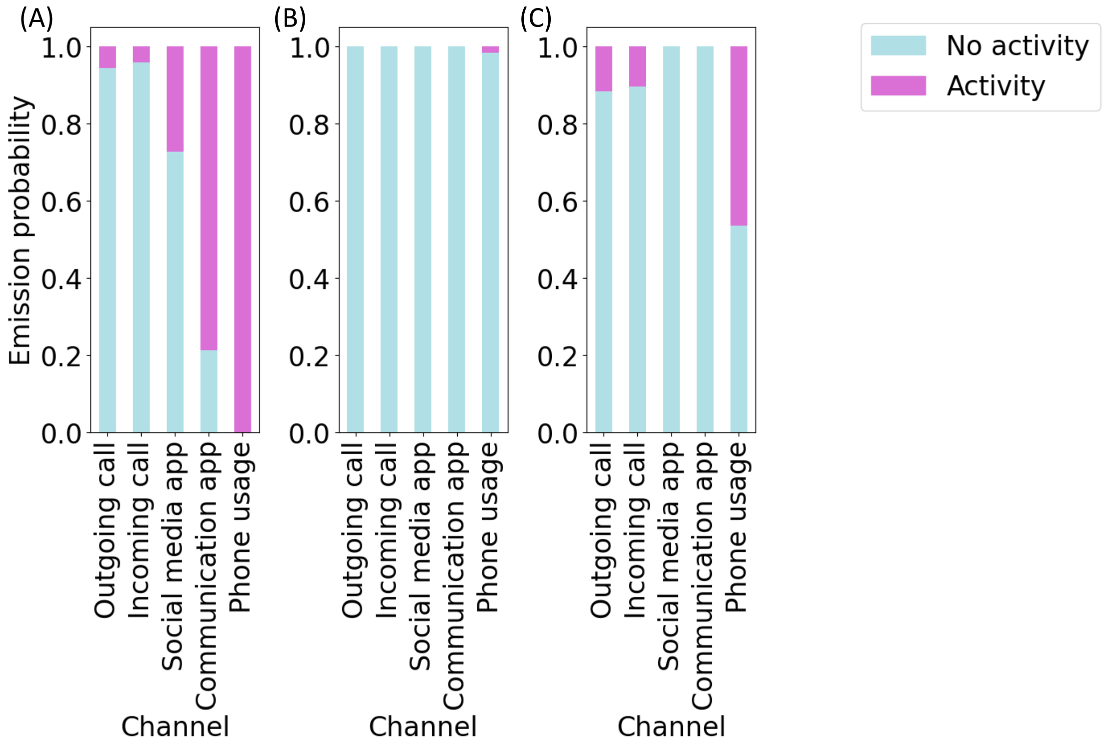

Figure S7: Emission probabilities for three state HMM trained using all data, including hour as a covariate, for (A) state 1, (B) state 2 and (C) state 3.

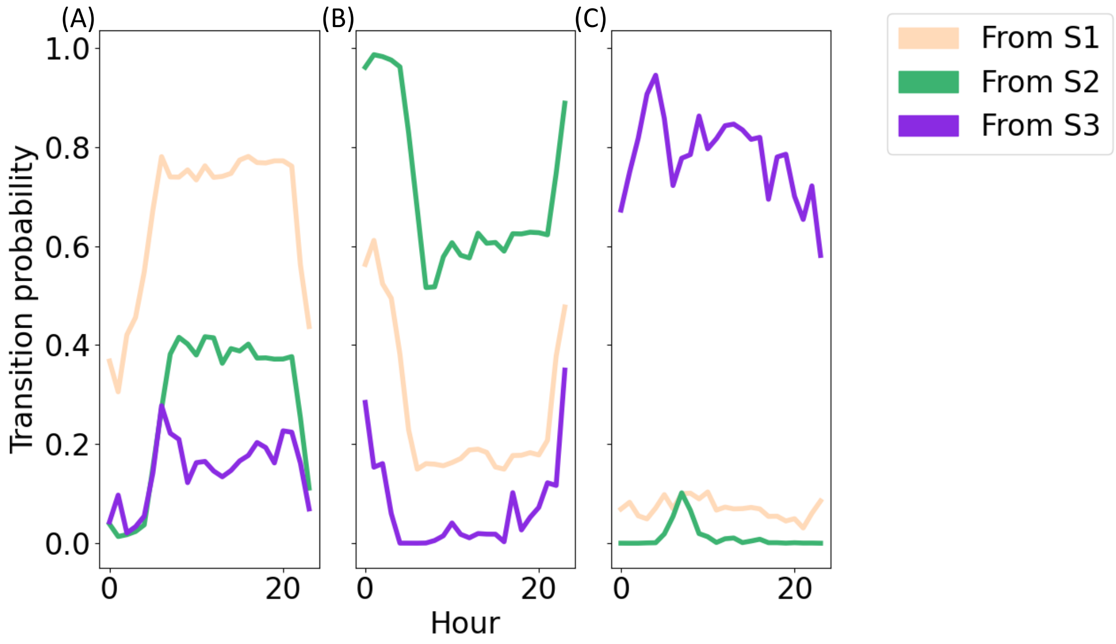

Figure S8: Transition probabilities for three state HMM trained using all data, including hour as a covariate, reflecting transitions to (A) state 1, (B) state 2 and (C) state 3.

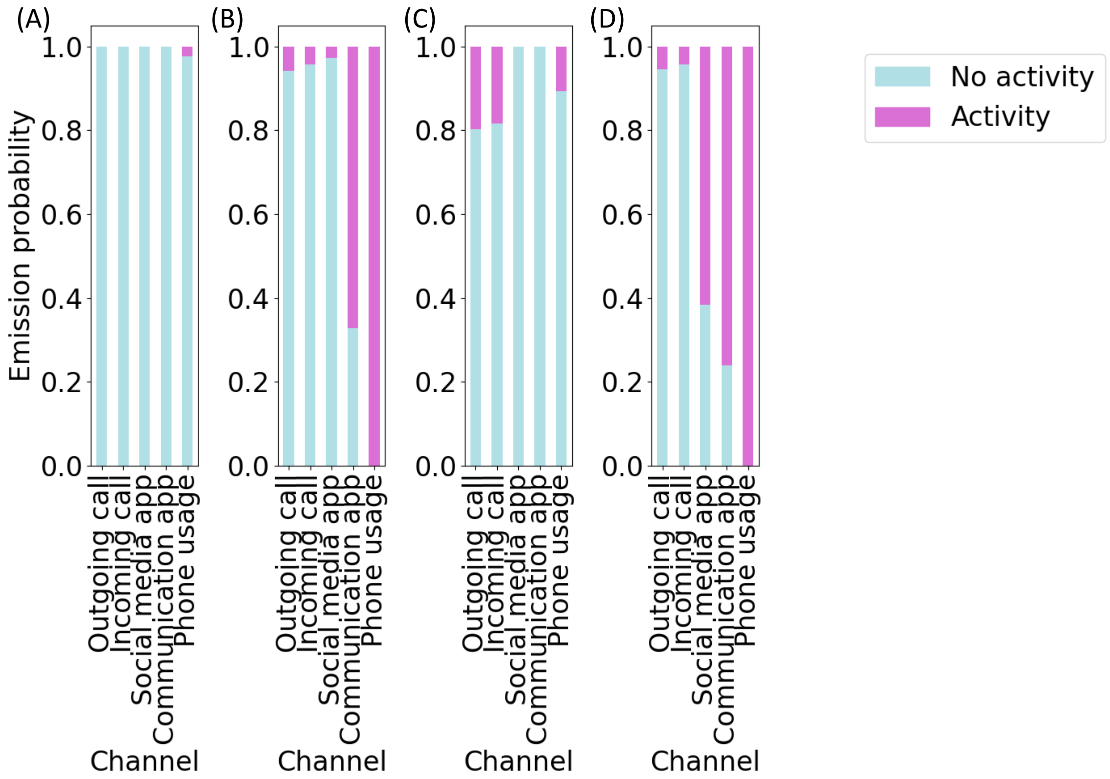

Figure S9: Emission probabilities for four state HMM trained using all data, including hour as a covariate, for (A) state 1, (B) state 2, (C) state 3 and (D) state 4.

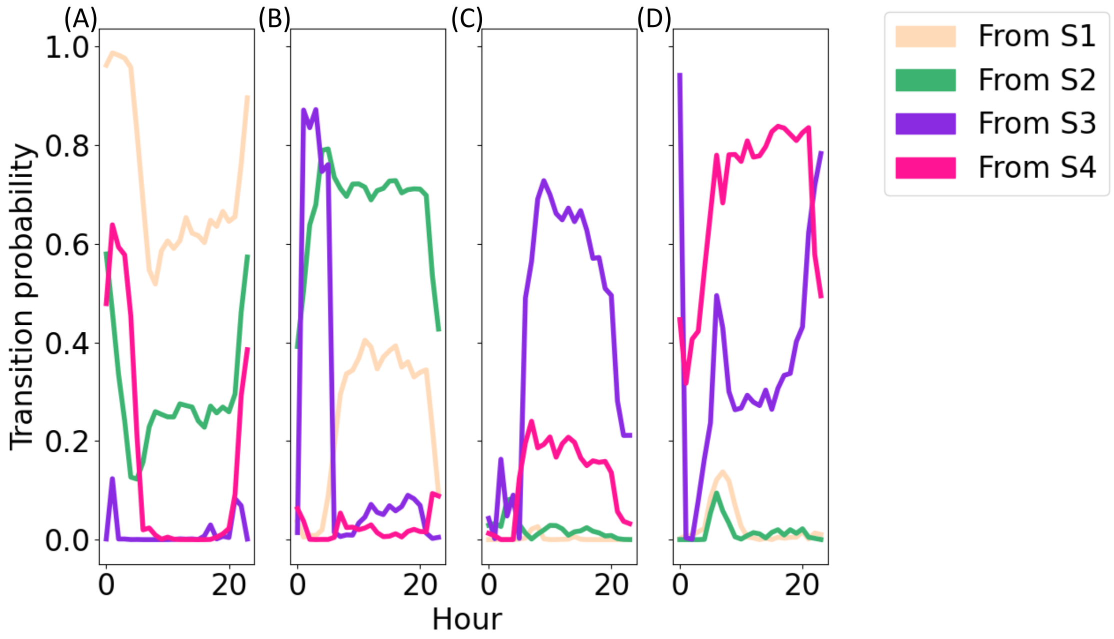

Figure S10: Transition probabilities for four state HMM trained using all data, including hour as a covariate, reflecting transitions to (A) state 1, (B) state 2, (C) state 3 and (D) state 4.

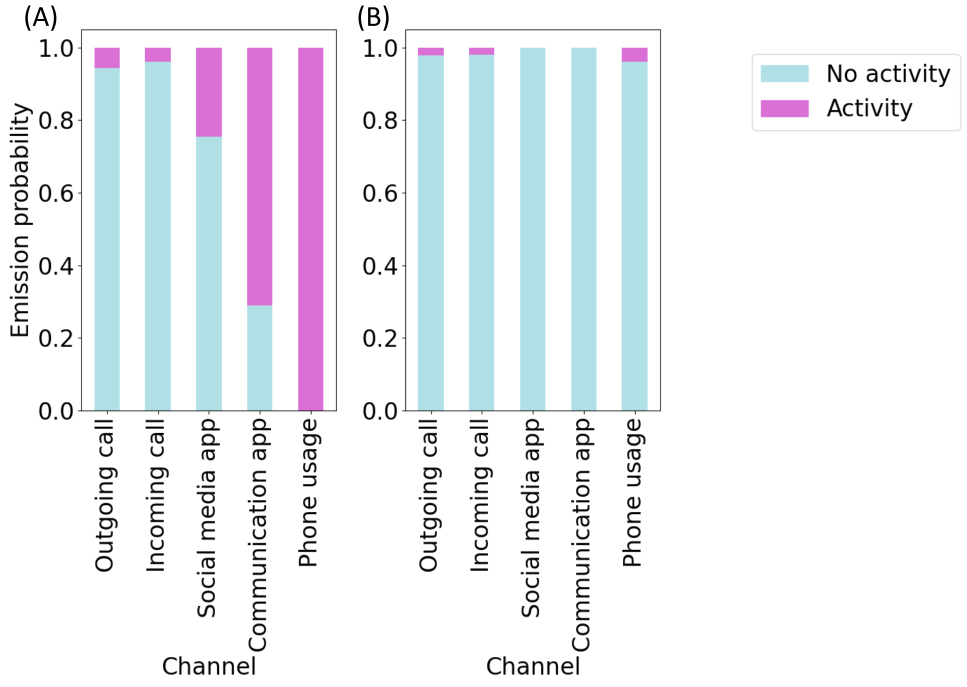

Figure S11: Emission probabilities for two state HMM trained using all healthy controls, including hour as a covariate, for (A) state 1 and (B) state 2.

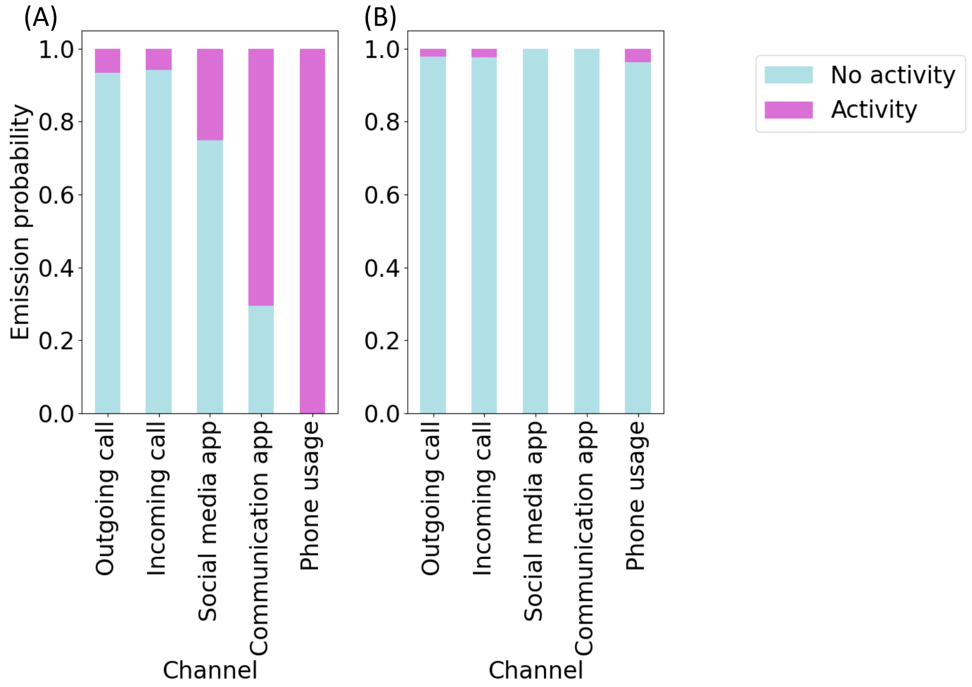

Figure S12: Emission probabilities for two state HMM trained using all diagnostic groups, including hour as a covariate, for (A) state 1 and (B) state 2.
